# MEK5/ERK5 inhibition sensitizes *NRAS*-mutant melanoma to MAPK-targeted therapy by preventing Cyclin D/CDK4-mediated G1/S progression

**DOI:** 10.1038/s41419-025-08036-7

**Published:** 2025-10-06

**Authors:** Rupesh Paudel, Simon Goller, Felix Deutzmann, Alina Gillitzer, Katharina Meder, Andrea Knorz, David Schrama, Matthias Goebeler, Marc Schmidt

**Affiliations:** Department of Dermatology, Venereology and Allergology, University Hospital Würzburg, Würzburg, Germany

**Keywords:** Melanoma, Cancer models, Stress signalling, Melanoma

## Abstract

Despite the advent of immune-oncological therapies, patients with advanced *NRAS*-mutant melanoma still have a significantly worse prognosis than their *BRAF*-mutant counterparts. This is mainly due to a high propensity for resistance to available therapies targeting the RAS/RAF/MEK/ERK mitogen-activated protein kinase (MAPK) pathway (MAPKi). Preclinical studies and mouse models have implicated the stress-activated MEK5/ERK5 MAPK cascade as a major resistance pathway activated by MAPKi-based targeted therapy in *NRAS*-mutant melanoma. Accordingly, MAPKi/ERK5i co-inhibition was capable of triggering a sustained cell cycle arrest in *NRAS*-mutant melanoma cells, but the key mediator(s) of its vigorous anti-proliferative effect remain elusive. Here, we further investigated the mechanism of MAPKi/ERK5i-induced cell cycle arrest in *NRAS*-mutant melanoma cells using both genetic methods and pharmacological inhibitors. Transcriptome analysis of human *NRAS*-mutant melanoma cells established that MAPKi/ERK5iinduced a near-complete shutdown of the mitotic machinery as consequence of a sustained G1 cell cycle arrest. This arrest was not only observed in diverse treatment-naïve melanoma cells but could also be induced in cells that already had developed resistance to therapeutic MEK inhibition (MEKi) and was accompanied by suppression of Cyclin D1 and E2F-mediated gene expression. Forced expression of Cyclin D1 and its effector kinase CDK4 restored cell cycle progression and mitotic gene expression in *NRAS-*mutant melanoma cells exposed to MEKi/ERK5i, implying Cyclin D/CDK4 activity as major target of combined MEKi/ERK5i. These findings suggest Cyclin D/CDK4 dependency as a major vulnerability of *NRAS*-mutant melanoma that could effectively be targeted by combined MAPKi/ERK5i.

## Introduction

Cutaneous melanoma accounts for the majority of skin cancer-related mortality [1]. A typical feature is the oncogenic activation of *BRAF* ( ~ 50%) or *NRAS* ( ~ 20-30%), hyper-activating the MEK1/2-ERK1/2 mitogen-activated protein kinase (MAPK) cascade [2]. Besides immune checkpoint blockade, MAPK inhibition (MAPKi) by pharmacological inhibitors of BRAF and MEK1/2 (MEKi) thus is a valuable treatment [3]. Particularly patients with advanced *BRAF*-mutant melanoma profit from a rapid initial response, but MAPKi also remains effective for individuals not responding, progressing or experiencing adverse events under immune checkpoint blockade. However, MAPKi-based therapy is prone to resistance development [4] and not sufficiently effective in NRAS-mutant melanoma due to frequent intrinsic and acquired resistance [5].

The stress-activated MEK5-ERK5 MAPK cascade has recently gained much attention as escape route for various cancer entities with MAPK activation, allowing them to survive and proliferate under MAPKi [6]. Particularly in RAS-driven tumors, combined MAPKi with ERK5i markedly improved MAPKi-based therapies, partly by preventing therapy resistance and switching to ERK5-mediated proliferation [7–10].

We have previously demonstrated that MEKi results in ERK5 activation and that concomitant MEK/ERK5 inhibition induces apoptosis and prolonged cell cycle arrest in both *BRAF*- and *NRAS*-mutant melanoma [7]. This suggests ERK5 co-inhibition as treatment option, but its mode of action and molecular effectors remain unclear.

Here, we performed RNA-sequencing (RNA-seq) of human melanoma cells exposed to the MEKi Trametinib (Tram) or Tram combined with siRNA targeting the ERK5-activating kinase MEK5 to elucidate the benefit of combined MEKi/ERK5i versus MEKi alone. As major value of combined MEKi/ERK5i treatment, we identified the induction of a sustained G1-arrest that occurs at the level of Cyclin D1/Cyclin-Dependent Kinase 4 (CDK4) activity, leading to secondary shutdown of the mitotic machinery in both naïve and MEKi-resistant melanoma cell lines. Ectopic Cyclin D1/CDK4 expression restored G1/S progression, validating Cyclin D/CDK4 as a key target of combined MEKi/ERK5i and a common hub of cell cycle regulation by both MAPK pathways.

## Results

### RNA-seq reveals a role of MEK5 in proliferation under MEKi

**Fig. 1:**
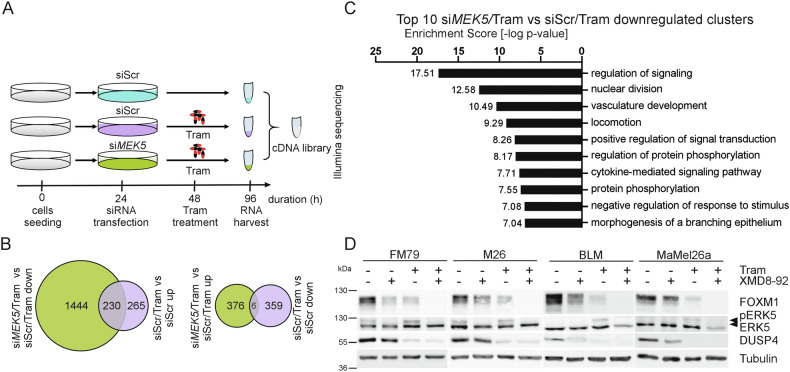
RNA sequencing uncovers efficient cell cycle inhibition as main benefit of combined MEKi/ERK5i. **A:** Treatment scheme and timeline of RNA-seq analysis using *NRAS*-mutant FM79 melanoma cells. Samples from *n* = 3 independent experiments with the illustrated conditions were used for statistical evaluation. **B:** Venn-diagram, displaying the numbers of ≥ 2.0-fold statistically (*p*_adjusted_ < 0.05) up- or downregulated genes found upon comparison of the si*MEK5*/Tram and Tram/siScr conditions (green) or the Tram-exposed and unstimulated siScr-transfected samples (violet). **C:** List of the 10 topmost enriched functional clusters identified for the statistically ≥2.0-fold downregulated genes comparing Tram/si*MEK5-* vs Tram/siScr-treated FM79. Statistical enrichment in relation to all genes expressed in the human genome was determined by DAVID-functional annotation cluster analysis [12] using the gene ontology biological process functional annotation term assignments of the genes as analysis criterion. Designated functional clusters were ranked according to their enrichment scores defined as negative logarithm of the collective mean *p*-values of the included gene ontology biological process functional annotation terms as calculated by Fisher’s exact test. **D:** Representative immunoblots from *n* = 3 independent experiments, showing efficient suppression of the G2M master regulator FOXM1 upon two days of co-treatment with Tram and the ERK5i XMD8-92 in each two different *NRAS*-mutant melanoma cells with (FM79 and M26) and without (BLM and MaMel26a) basal ERK5 autophosphorylation activity. ERK5 autophosphorylation and its suppression by XMD8-92 are reflected by presence or absence of a slower migrating band. For validation of effective Tram activity and equal loading, additional immunoblots for the established ERK1/2 target DUSP4 and Tubulin are shown. The Tram concentrations used in FM79, M26, BLM and MaMel26 were 5, 2.5, 25, and 5 nM, respectively. XMD8-92 was used at 5 µM irrespective of the cell line.

To uncover the benefit of additional ERK5i versus MEKi alone, we performed RNA-seq of untreated scrambled siRNA (siScr)-transfected FM79 melanoma cells and FM79 transfected with either siScr or *MEK5* siRNA and subsequently exposed to 5 nM Tram for 48 h (Fig. 1A). FM79 cells have an NRAS^Q61L^ driver mutation and show basal ERK5 phosphorylation, rendering them MEKi- and ERK5i-responsive but particularly susceptible to their combination [7]. This allowed us to distinguish between genes affected by Tram or *MEK5* siRNA alone and those regulated by Tram in a MEK5-dependent manner. Functionality controls confirmed efficient *MEK5* knockdown and mRNA repression of the ERK1/2 target *DUSP4* [11] while principal component analysis validated homogeneity among replicates but heterogeneity between the samples (Supplementary Fig. 1A–C).

Analysis of the ≥ 2-fold differentially expressed genes (DEGs) revealed ~2.5-fold more DEGs (2056 vs 860) in the Tram/si*MEK5* co-treated condition compared to Tram alone, with almost 80% of DEGs downregulated (Fig. 1B). Moreover, ∼50% of the Tram-induced transcriptional response was mediated by MEK5/ERK5 since almost half (230/495) of the Tram-induced DEGs were significantly suppressed upon si*MEK5* co-treatment (Fig. 1B). Functional annotation cluster analysis using the publicly available Database for Annotation, Visualization, and Integrated Discovery (DAVID) [12], identified “*nuclear division*” encompassing 96 cell cycle-related genes (Supplementary Table 1) as the second most overrepresented functional term among the si*MEK5*-downregulated genes (Fig. 1C). Moreover, we found enrichment of several other mitosis-related clusters, suggesting that *MEK5* depletion affects mitotic gene expression. Interestingly, we spotted the transcription factor *FOXM1* (Fold change = -2.92, *p* = 2.80e^-12^), a master regulator of G2M progression [13, 14] and several of its transcriptional targets such as *CCNB1*, *PLK1* and *AURKB* among the si*MEK5-*suppressed genes (Supplementary Fig. 1D, E). Since this suggested FOXM1 as a relevant target of combined MEKi/ERK5i, we compared the impact of Tram monotreatment or its combination with the ERK5i XMD8-92 [15] on FOXM1 protein levels for two *NRAS*-mutant melanoma cell lines with (FM79 and M26) or without (BLM and MaMel26a) basal ERK5 phosphorylation [7]. Irrespective of ERK5 phosphorylation status, Tram/XMD8-92 co-administration reduced FOXM1 levels more efficiently than the monotreatments (Fig. 1D).

### MEKi/ERK5i-dependent mitotic gene suppression is secondary to G1-arrest

**Fig. 2:**
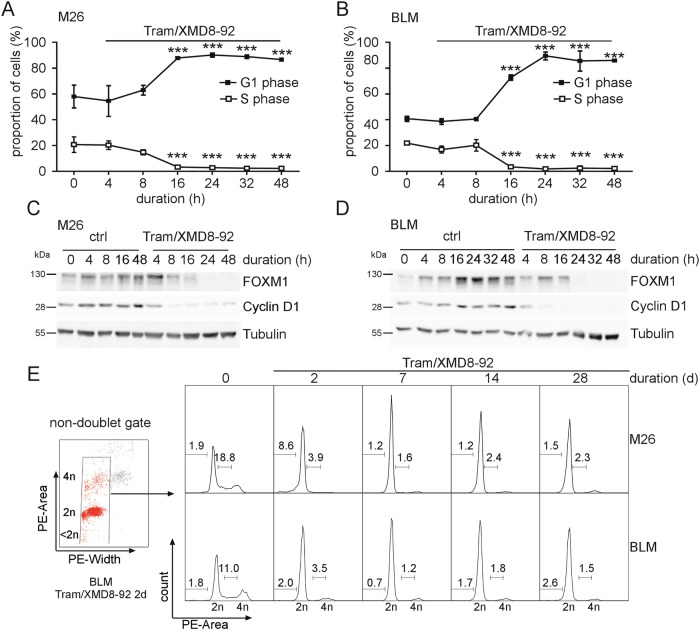
Kinetics of MEKi/ERK5i-induced G1-arrest in melanoma cells. **A**, **B** Flow cytometric cell cycle analysis of the indicated *NRAS*-mutant melanoma cell lines upon co-treatment with Tram and XMD8-92 as determined by propidium iodide (PI)-mediated DNA profiling. Line diagrams showing percentages of cells in G1- and S-phase as a function of time for M26 (**A**) or BLM (**B**). Data are represented as mean values ± SD from three independent experiments. Statistically significant differences to the respective untreated value at 0 h are indicated by asterisks (***p < 0.001, one-way Anova with post-Dunnet test). **C, D** Representative immunoblots, of *n* = 2 independent experiments, showing expression of Cyclin D1 and FOXM1 proteins under MEKi/ERK5i combination treatment for the indicated times in M26 (**C**) or BLM (**D**). **E** Long-term effect of Tram/XMD8-92 on cell cycle distribution of M26 (top) or BLM (bottom). Histograms are representative of *n* = 2 experiments and show DNA profiles obtained by flow cytometric analysis of PI-stained cells with calculated percentages of sub-G1 and S-phase cells obtained after PE-A/PE-W doublet discrimination as shown on the left. In all experiments, cells were treated with 2.5 (M26) or 25 nM Tram (BLM) and 5 µM XMD8-92. In (**E**) medium was replaced every two to three days by fresh medium supplemented with diluent or the respective inhibitors and except for the two-days treatment, cells were reseeded at equal density into fresh inhibitor- or diluent-containing medium the day before harvesting.

Loss of mitotic gene expression could either be a direct effect of MEKi/ERK5i co-treatment or a secondary event to an arrest at earlier phases of the cell cycle. To differentiate between both possibilities, we performed DNA profiling kinetics for representative *NRAS*-mutant melanoma cell lines with (M26) or without (BLM) basal ERK5 phosphorylation. We found no impaired G2M progression but observed a rapid G1-arrest after 16 h of Tram/XMD8-92 co-treatment (Fig. 2A, B and Supplementary Fig. 2A). The arrest correlated with decreased mRNA and protein levels of the key G1/S regulator Cyclin D1 [16], which dropped as early as 8 h after co-treatment, while FOXM1 reduction was delayed (Fig. 2C, D and Supplementary Fig. 2B, C). Thus, mitotic gene suppression occurred secondarily to G1/S inhibition. Moreover, the suppression of G1/S progression was independent of the phenotypic status as all four melanoma cell lines tested exhibited different phenotypic differentiation status as indicated by distinct expression of phenotypic markers such as MITF, SOX10, AXL, E-Cadherin, N-Cadherin, ZEB1 and ZEB2 (Supplementary Fig. 2D).

We next re-examined our RNA-seq data for presence of G1/S-regulators that could have been masked by our initial threshold ( ≥ 2-fold change, *p* < 0.05). Re-sorting our original lists by *p*-value (*p* < 0.05) and combining fold changes by Tram and Tram/si*MEK5* treatment we observed enhanced suppression of several G1/S genes by the combination (Supplementary Table 2), suggesting that ERK5i primarily augments and sustains the anti-proliferative effects of MEKi. Both DAVID analysis of significantly downregulated genes and BrdU labeling experiments supported this view. Indeed, clusters related to the cell-cycle demonstrated the highest enrichment scores for the Tram- and si*MEK5*-co-suppressed genes (Supplementary Table 3, compare with Fig. 1C). Accordingly. BrdU incorporation was further decreased upon Tram/XMD8-92 co-treatment compared to Tram alone (Supplementary Fig. 3). Two genes frequently appearing in the top enriched clusters were *CCND1* and the E2F target *FBXO5* (also known as Early Mitotic Inhibitor, EMI1). While Cyclin D1 initiates inactivation of Retinoblastoma (RB)-mediated E2F suppression, EMI1 serves both as a substrate and inhibitor of the Anaphase-Promoting Complex/Cyclosome (APC/C^cdh1^) and switches off APC/C^cdh1^ proteasomal activity in late G1 stage enabling accumulation of various cell cycle proteins once E2F activity increases [16]. This ensures progression through the restriction point and irreversible commitment to S-phase [17]. The co-regulation of *CCND1*, *FBXO5* and other E2F targets indicated an arrest prior to E2F activation by stabilizing RB function. Indeed, large-scale promoter analysis of our Tram/si*MEK5*-co-suppressed DEGs using the publicly available tool PSCAN [18] revealed a strong overrepresentation of several E2F transcription factors and their binding partner TFDP1 (Supplementary Table 4). Consistently, we could validate a sustained Tram/XMD8-92-induced G1-arrest in M26 and BLM (Fig. 2E). Moreover, as previously noted, the antiproliferative response dominated over apoptosis in these two cell lines [7] and sub-G1 content remained marginal, regardless of whether analysis was performed with or without exclusion of the sub-G1 fraction (compare Fig. 2E and Supplementary Fig. 2A, 48 h timepoints). All following cell cycle analyses were therefore performed excluding sub-G1 cells.

### MEKi-resistant melanoma cells are still sensitive to combined MEKi/ERK5i

**Fig. 3:**
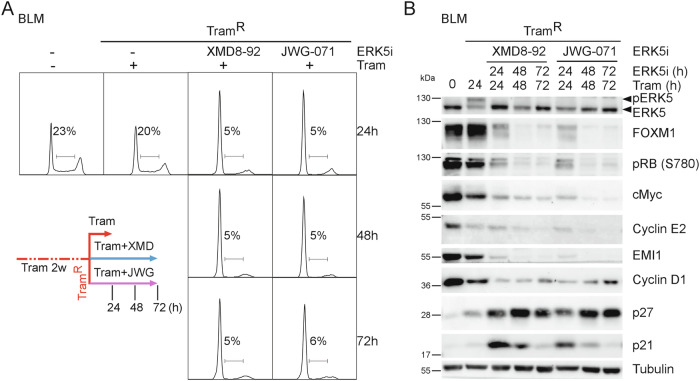
ERK5i co-treatment can restore G1-arrest in Tram^R^ melanoma cells. BLM cells were pre-treated with Tram for three weeks and re-seeded into Tram-containing medium. The next day, the cells were supplemented with diluent or the indicated ERK5i (XMD8-92 or JWG-071; 5 µM each) in addition to Tram for the specified time periods. **A** Representative DNA profiles from *n* = 3 independent experiments. Calculated percentages of S-phase cells are shown for each condition. Colored lines indicate the treatment scheme. Corresponding quantifications are shown in Supplementary Fig. 4C and D. **B** Representative immunoblots of *n* = 3 experiments, illustrating altered expression of the indicated cell cycle-relevant proteins in total cell lysates. An immunoblot for ERK5 using an antiserum detecting both its non-phosphorylated and autophosphorylated form is included as functionality control for Tram and the employed ERK5i. Tubulin served as loading control.

Acquired MEKi resistance is mediated by activation of compensatory pathways including receptor tyrosine kinases [19–21], which in part can activate the MEK5/ERK5 pathway [7]. To check whether ERK5i could still arrest Tram-resistant melanoma cells that had already escaped the anti-proliferative effect of primary MEKi, we first investigated at which time-point Tram-treated BLM re-initiated proliferation. Supplementary Fig. 4A illustrates that Tram-treated cells re-entered the cell cycle after one week of treatment and proliferated stably after two to four weeks. Drug tolerance of melanoma cells during MAPKi resistance frequently correlates with phenotypic switching towards hyperdifferentiated MITF^high^/SOX10^high^ or invasive/dedifferentiated MITF^ow^/SOX10^low^ phenotypes [22, 23]. For the BLM cell line, we failed to observe such changes (Supplementary Fig. 4B), presumably because drug naïve BLM already display an invasive de-differentiated phenotype (Supplementary Fig. 2D). Interestingly, when we subjected the Tram-pretreated, proliferating BLM cells (hereafter referred to as “Tram-resistant” (Tram^R^) cells) to Tram/ERK5i (XMD8-92 or JWG-071 [24]) co-treatment for 24 to 72 h, they regained G1-arrest within 24 h, as indicated by significantly reduced proportion of S-phase cells (Fig. 3A), and concomitant accumulation of G1 cells (Supplementary Fig. 4C, D). Parallel immunoblots validated Tram-induced ERK5 auto-phosphorylation and its suppression by ERK5i within 24 h. At this time-point, we also observed loss of RB-monophosphorylation at Serine 780 (S780), a Cyclin D/CDK4 specific site [25], and reduced expression of Cyclin D1, cMyc, and the E2F targets Cyclin E2 and EMI1. Moreover, we confirmed a progressive loss of FOXM1 protein (Fig. 3B), which again occurred secondarily to the induced G1-arrest, as *CCND1* mRNA was significantly reduced after 8 h of XMD8-92 or JWG-071 co-exposure, while neither *FOXM1* nor *CCNE2* mRNA were significantly reduced (Supplementary Fig. 4E). Additionally, we observed upregulation of p27 and p21 at the protein level (Fig. 3B). Notably, p21 upregulation was only transient, while p27 expression steadily increased over time. Thus, ERK5i can also trigger a G1-arrest in MEKi-resistant melanoma cells by modulating expression of key G1/S regulators.

### MEK5 is required for G1/S progression under MEKi

**Fig. 4:**
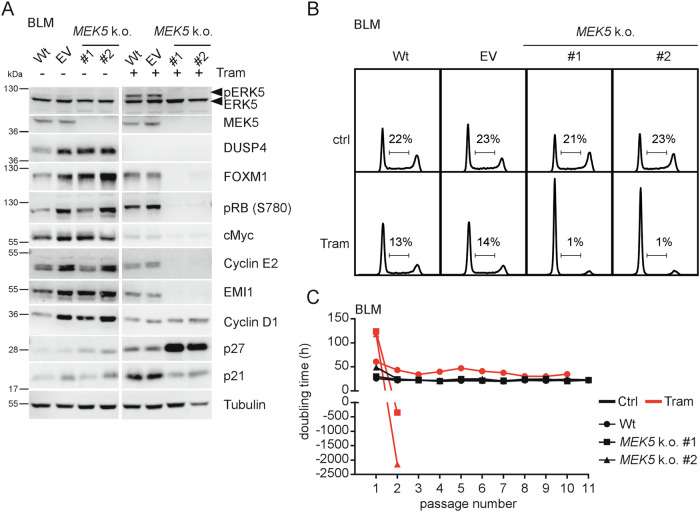
MEK5 is dispensable for normal proliferation of *NRAS*-mutant melanoma cells but required for G1/S progression under MEKi. **A**: Representative immunoblots of *n* = 3 experiments done using total lysates from BLM wild-type (Wt), BLM empty vector (EV)-infected, or two different CRISPR/*Cas9*-mediated *MEK5* k.o. single cell clones (SCC) of BLM treated with diluent (ctrl) or 25 nM Tram for 14 days, showing expression of selected cell cycle proteins. ERK5 phosphorylation and DUSP4 suppression confirm Tram functionality. Successful *MEK5* gene disruption was analysed by MEK5 immunoblot and functionally evaluated by immunoblotting for ERK5, which confirmed absent ERK5 autophosphorylation. Tubulin served as loading control. **B**: Representative cell cycle profiles of *n* = 2 experiments, as determined by flow cytometric analysis of the indicated PI-stained conditions with percentages of S-phase cells indicated. **C**: Cell doubling time analysis representative of *n* = 2 experiments with running times of 2–5 weeks performed with Wt BLM or the indicated *MEK5* k.o. BLM SCCs cultured in absence or presence of Tram. The shown experiment run over a period of five weeks.

We next employed CRISPR/*Cas9*-mediated gene disruption to investigate if *MEK5* deficiency can reproduce the results obtained with pharmacological ERK5i. For this, we generated two *MEK5*-deficent single-cell clones (SCCs) that lacked the ability to induce Tram-mediated ERK5 phosphorylation, validating loss of *MEK5* function (Fig. 4A). Consistent with absent basal ERK5 phosphorylation in BLM, protein analysis of selected G1/S regulators, DNA profiling, and cell-doubling analysis showed no overt defect in cell cycle progression in treatment-naïve *MEK5* k.o. cells compared to parental BLM (Wt) or the corresponding vector cell line (Fig. 4A–C). However, Tram-treatment resulted in a robust G1-arrest of both *MEK5* k.o. SCCs, as indicated by loss of RB-S780 phosphorylation, decreased expression of Cyclin E2, EMI1, cMyc, and FOXM1 protein after two weeks of exposure, and drastically reduced S-phase distribution in DNA profiling experiments (Fig. 4A, B). As observed with pharmacological ERK5i, we also found increased expression of p27 but no p21 induction by Tram (Fig. 4A). Consistent with our earlier results with Tram/XMD8-92 [7], cell-doubling time analysis further revealed negative doubling times for both *MEK5* k.o. SCCs, indicating progressive cell loss upon five weeks of Tram-treatment. Instead, untreated or Tram-treated Wt BLM could be passaged >10 times during this period (Fig. 4C). Accordingly, MEK5 is dispensable for normal proliferation of *NRAS*-mutant melanoma cells but critical for G1/S progression under MEKi.

### Cyclin D1/CDK4 overexpression alone is insufficient to fully overcome the G1-arrest induced by MEKi/ERK5i co-treatment

**Fig. 5:**
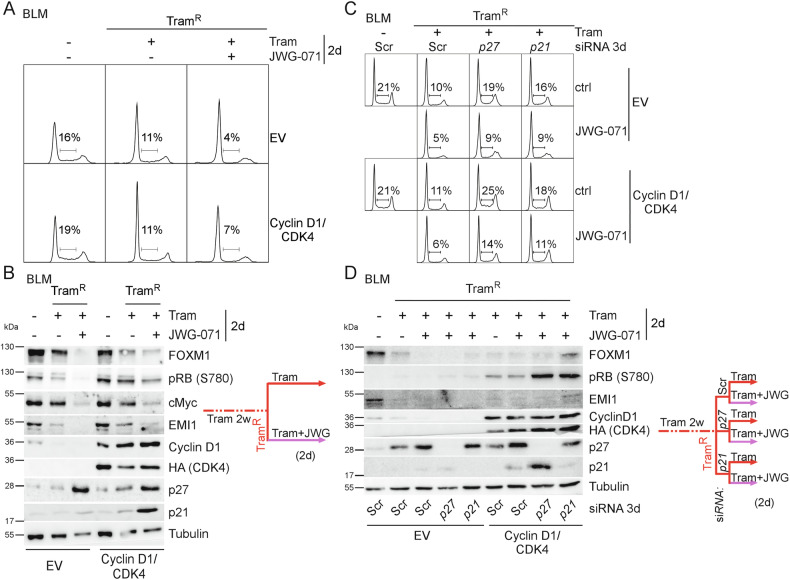
Ectopic CyclinD1/CDK4 expression alone is insufficient to fully revert the anti-proliferative effects of short-term ERK5i co-treatment on Tram^R^ cells. **A**: Cell cycle profiles of empty vector (EV)- or stably Cyclin D1/CDK4-expressing BLM after acquisition of Tram resistance (Tram^R^) by five weeks of Tram pre-treatment and subsequent Tram exposure with or without 5 µM of the ERK5i JWG-071 for two days. Histograms are representative of *n* = 3 independent experiments. Numbers denote the percentage of cells in S-phase. Quantifications are shown in Supplementary Fig. 5A. **B**: Representative immunoblots from *n* = 3 experiments, for the indicated cell cycle-relevant proteins from total cell lysates harvested in parallel to **A**. Tubulin served as loading control. **C**: Representative cell cycle profiles from *n* = 3 EV- or stably Cyclin D1/CDK4-expressing BLM after acquisition of Tram^R^ by five weeks of Tram-pre-exposure and a subsequent two-day Tram stimulation with or without (ctrl) the ERK5i JWG-071 following transfection with the indicated siRNA. Profiles of untreated siScr-transfected BLM with the indicated genetic manipulation are included for comparison. Quantifications are shown in Supplementary Fig. 5E. **D**: Corresponding representative *n* = 3, immunoblots for relevant cell cycle proteins from total cell lysates of an experiment performed in parallel to **C**. Colored lines illustrate the treatment scheme.

To pinpoint the G1/S regulators mediating the G1-arrest, we performed rescue experiments. Considering that Cyclin D/CDK4 activation is crucial for G1/S progression and FOXM1 stabilization [26], we suspected Cyclin D1 as the most likely target. Hence, we ectopically expressed Cyclin D1 and its kinase CDK4 as HA-tagged fusion protein and investigated whether they could overcome the Tram/ERK5i-imposed G1-arrest. In short-term experiments lasting two to three days, overexpression did not show any proliferation advantage (data not shown). However, in Tram^R^ cells, Cyclin D1/CDK4 expression resulted in a mild rescue of the ERK5i-induced G1-arrest, as indicated by partial recovery of S-phase progression in DNA profiling experiments 48 h after MEKi/ERK5i co-exposure (Fig. 5A and Supplementary Fig. 5A). Compared to the vector cell line, we further observed a moderate increase of RB-S780 phosphorylation, EMI1, and FOXM1 protein expression under short-term Tram/JWG-071 co-treatment. Nevertheless, expression of key cell cycle proteins did not reach the same level as in cells maintained under Tram alone (Fig. 5B), suggesting a possible contribution of other targets. We obtained similar results when Cyclin D1/CDK4-overexpressing cells were concurrently treated for two weeks (Supplementary Fig. 5B–D). Consistently, p21 and p27 proteins were likewise upregulated.

To explore an additional contribution of p21 and p27, we pre-exposed Cyclin D1/CDK4-overexpressing BLM and the corresponding vector cell line to Tram for two weeks, and transfected the resulting Tram^R^ cell lines with scrambled siRNA, *p21* or *p27* siRNA prior to Tram/diluent or Tram/JWG-071 co-administration for two days. Irrespective of Cyclin D1/CDK4 expression, depletion of p21 or p27 neutralized the residual anti-proliferative effect of Tram-treatment. However, reversal of the additional antiproliferative effect of JWG-071 co-treatment was only efficient when *p27* or *p21* knockdown was combined with Cyclin D1/CDK4 overexpression (Fig. 5C and Supplementary Fig. 5E). Parallel immunoblots confirmed the functionality of the employed siRNAs and validated a partial restoration of key cell cycle proteins, including phospho-RB-S780, EMI1, and FOXM1 expression (Fig. 5D) upon combined Cyclin D1/CDK4 expression and *p21* or *p27* siRNA. These results indicate that the MEKi/ERK5i-induced arrest is robust and withstands modulation of individual G1/S regulators.

### Cyclin D1/CDK4 re-expression is sufficient to restore G1/S progression in long-term MEKi/ERK5i arrested cells

**Fig. 6:**
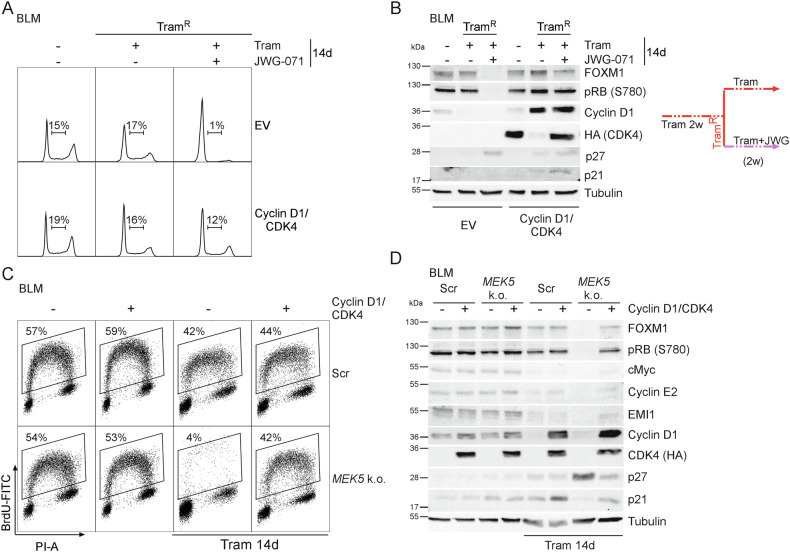
Ectopic Cyclin D1/CDK4 expression alone is sufficient to overcome the long-term anti-proliferative effects of subsequent combined MEKi/ERK5i exposure. **A**: Representative cell cycle profiles from *n* = 3 EV- or stably Cyclin D1/CDK4-expressing BLM after acquisition of Tram resistance (Tram^R^) by two weeks of Tram-pretreatment and subsequent co-treatment with Tram and 5 µM of the ERK5i JWG-071 for additional two weeks. Numbers denote the calculated percentage of S-phase cells for the respective DNA profile. Quantifications are shown in Supplementary Fig. 6A upper panel. **B**: Representative immunoblots *n* = 3 for the indicated cell cycle-relevant proteins from total cell lysates harvested in parallel to **A**. **C**: Representative dot plots from *n* = 2 BrdU labelling experiments showing the percentage of BrdU-positive cells following the stable expression of an empty vector (-) or Cyclin D1/CDK4 in either scrambled gRNA (Scr)-infected or *MEK5* k.o. BLM cells and treatment with Tram for 14 days. **D**: Corresponding immunoblots *n* = 3, for selected cell cycle-relevant proteins with total cell lysates of a parallel experiment to **C**. Colored lines in **B** and **D** indicate the respective treatment schemes.

Ectopic Cyclin D1/CDK4 expression could only partially reverse the anti-proliferative effect of short-term Tram/ERK5i co-treatment of Tram^R^ BLM or two-week-co-treatment of treatment-naïve BLM. We thus explored whether ectopic Cyclin D1/CDK4 could restore cell cycle progression if ERK5i/MEKi-exposed cells were given sufficient time to adapt to *MEK5* deficiency or Tram exposure prior to co-treatment. To address this, freshly generated Tram^R^ cells were cultured in presence of Tram alone or Tram plus JWG-071 for additional 14 days. In this setting, Cyclin D1/CDK4 expression alone significantly improved cell cycle progression of the Tram/ERK5i co-treated cells as indicated by DNA profiling (Fig. 6A, Supplementary Fig. 6A top), restoration of RB-S780 phosphorylation and re-expression of FOXM1 in immunoblots (Fig. 6B). Parallel time-lapse-microscopy validated that Tram^R^ cells exposed to long-term Tram/JWG-071 treatment did indeed undergo mitosis and cytokinesis after stable Cyclin D1/CDK4 expression (Supplementary Movie 1). Crystal violet staining further confirmed that ectopic Cyclin D1/CDK4 expression conferred resistance to MEKi/ERK5i treatment in this setting (Supplementary Fig. 6A bottom).

Similarly, ectopic Cyclin D1/CDK4 expression prevented Tram-induced cell cycle arrest in our stable *MEK5* k.o. SCCs as indicated by restoration of S-phase progression (Fig. 6C, Supplementary Fig. 6B), time-lapse microscopy (Supplementary Movie 2), crystal violet staining (Supplementary Fig. 6C bottom), and re-initiation of RB-S780 phosphorylation, Cyclin E2, EMI1, and FOXM1 expression (Fig. 6D). In successive treatment regimen, Cyclin D1/CDK4 alone therefore is sufficient to overcome the MEKi/ERK5-induced G1-arrest while co-exposure of treatment-naïve cells produces a more robust G1-arrest.

## Discussion

Data from various tumor cell types with constitutive ERK activation imply that MAPKi/ERK5i-based therapy could significantly lower the risk of MAPKi resistance (reviewed in refs. [6, 27–29]) as recently also shown for a novel single molecule inhibitor for ERK1/2 and ERK5 in preclinical breast carcinoma models [30]. Here we investigated *NRAS*-mutant melanoma cells, a particular MAPKi-resilient melanoma sub-type. In earlier preclinical models, we demonstrated that several *NRAS*-mutant melanoma cells exhibit increased sensitivity to combined MEKi/ERK5i treatment than to either of the monotreatments [7]. Our unbiased transcriptomic data now show that the added benefit of ERK5i in MEKi-treated cells primarily lies in improved cell cycle inhibition. Transcriptionally, this was evident by downregulation of several gene clusters associated with nuclear division and mitotic progression, a pattern reminiscent of quiescent cells [31, 32]. Genetic, pharmacological and kinetic data, demonstrated that the prominent shut-down of mitotic genes was a consequence of prolonged G1-arrest. Similarly, Zhang et al. reported induction of G1-arrest upon HER2/ERK5 co-inhibition [33] and this arrest likewise correlated with enhanced suppression of RB-S780 phosphorylation, a Cyclin D/CDK4-specific site [25]. However, based on their previous observation that conditional *Erk5* deletion in murine fibroblasts had no effect on Cyclin D/CDK4 activity but suppressed Cyclin E/CDK2 activity via p21 and p27 induction [34], the authors concluded that the HER2i/ERK5i-induced cell cycle arrest occurred via suppression of Cyclin E/CDK2 activity. A role of p21 has also been reported in *BRAF*-mutant melanoma cells where ERK5 knockdown increased p21 expression and induced senescence [35]. We also noticed increased p21 and p27 expression upon combined MEKi/ERK5i in our short-term experiments and both p21 and p27 knockdown improved Cyclin D1/CDK4-mediated cell cycle re-entry under short-term Tram/JWG-071 treatment of Tram^R^ BLM. However, at least p21 induction was transient and lost in our long-term experiments. Moreover, ectopic Cyclin D1/CDK4 expression alone restored cell cycle progression in Tram^R^ cells subjected to prolonged Tram/ERK5i co-treatment and prevented Tram-induced G1-arrest of MEK5-deficient SCCs, possibly due to adaptation to *MEK5* deficiency during the selection process. Notably, JWG-071-induced *CCND*1 mRNA reduction in Tram^R^ cells occurred faster than *CCNE2* silencing, excluding *CCNE2* suppression as primary trigger for the induced G1-arrest. Our data therefore indicate that the robust G1-arrest induced by combined MEKi/ERK5i in *NRAS*-mutant melanoma cells primarily relies on suppression of Cyclin D1/CDK4 activity. This is likely due to Cyclin D1 suppression, although increased p21/p27 induction may additionally restrain CDK4 activity. In agreement, Mulloy et al. have previously reported a role of ERK5 in Cyclin D1 expression [36]. Importantly, Cyclin D1/CDK4 activity can also phosphorylate and stabilize FOXM1 protein, which is required for transactivation of certain E2F-target genes, including *CCNE2*, and timely S-phase progression [26]. It is tempting to speculate that the observed progressive loss of FOXM1 protein may further strengthen the G1-arrest by disrupting the accumulation of E2F targets, including Cyclin E2.

Intriguingly, ERK5i co-administration could also induce a G1-arrest in Tram^R^ cells. In agreement, Mondru et al. reported that MEKi/ERK5i co-administration, but not MEKi monotreatment, effectively restored cell cycle arrest in BRAF inhibitor-resistant *BRAF*-mutant melanoma cells [37]. Moreover, a BRAF-mutant MEKi- resistant, undifferentiated melanoma cell line was also reported to be sensitive to ERK5 inhibition [38]. Interestingly, *NRAS*-mutant melanoma cells are highly sensitive to combination therapies of MEKi/CDK4i [39]. In a recent phase Ib/II clinical trial for treatment of *NRAS*-mutant melanoma, co-treatment of the MEKi binimetinib with the CDK4/6 inhibitor ribociclib showed response rates of ~20% and median overall survival rates of 11.3 months [40]. Given that ERK5 activation has also recently been identified as resistance pathway for combined MEKi/CDK4i treatment of *NRAS*-mutant melanoma [41], we envisage that combined MEKi/ERK5i may be a better approach to treating *NRAS*-driven melanoma, as it is predicted to be less prone to resistance development. Notably, our results emphasize the importance of considering both timing of MEKi/ERK5i treatment and CDK4 activation status for translational considerations since in sequential treatment regimens Cyclin D1/CDK4 expression alone was capable of overriding the MEKi/ERK5i-induced proliferation arrest. However, given the low rate of amplification/activating mutations in Cyclin D1/CDK4 in melanoma (∼5-7%) [42–44], resistance by primary CDK4 activation is a minor concern.

Overall, our data demonstrate that ERK5i can significantly enhance the efficacy of MEKi in *NRAS*-mutant melanoma. The induced G1-arrest is multifactorial and robust, potentially reducing the likelihood of resistance development. Moreover, it obviously is independent of the melanoma differentiation status. Our study further highlights the importance of proper timing of MEKi/ERK5i therapy narrowing the window for developing resistance. Given the robust and durable cell cycle arrest, we propose that particularly *NRAS*-mutant melanoma patients ineligible for or resistant to immune checkpoint blockade may benefit from MEKi/ERK5i combination therapies.

## Materials and methods

### Cell culture, treatments and generation of Tram-resistant cell lines

The *NRAS*-mutant melanoma cell lines FM79, BLM, MaMel26a and M26 have all been characterized earlier [45–48]. Cells were routinely tested for mycoplasma and grown at 37 °C with 5% CO_2_ in RPMI1640 GlutaMAX medium (ThermoFisher Scientific, Darmstadt, Germany), supplemented with 10% fetal calf serum (Capricorn, Ebsdorfergrund, Germany) and 30 µg/ml Gentamycin (#G1397, Sigma Aldrich, Darmstadt, Germany). The MEKi Tram was purchased from Enzo (#ENZ-CHM239; Lörrach, Germany) and used at 2.5 (M26), 5 (MaMel26a, FM79) or 25 nM (BLM), respectively. The ERK5 inhibitors, XMD8-92 (#HY-14443) and JWG-071 (#HY-108886) were purchased from MedChemTronica (Sollentuna, Sweden), and used at 5 µM.

Tram-resistant (Tram^R^) BLM were generated by growing drug-naïve cells in 25 nM Tram-containing media for a period of two to five weeks until they proliferated constantly. Pre-exposures were done freshly each time to allow acquisition of Tram resistance without preselection of single resistance mechanisms. For co-treatments, Tram^R^ cells were re-seeded into Tram-containing media at a density of 5 × 10^3^ cells/cm^2^ and exposed the following day to medium containing Tram alone or Tram and 5 µM ERK5i for one to three days or 14 days, with medium and inhibitor replacement every three to four days.

### Western blot

Western blots were performed as described [49] using the antibodies listed in Supplementary Table 5.

### Generation of *MEK5*-deficient cell lines by lentiviral CRISPR/*Cas9*

For generation of *MEK5* k.o. BLM, lentiCRISPR_zeo, a derivative of the lentiviral dual gRNA/*Cas9* expression vector lentiCRISPRv2-Blast (Addgene_83480) replacing the blasticidin^R^ gene by a zeocin^R^ gene was used. The scrambled gRNA (5’-ATTCAGCGCGCTCGCCCTGG-3’) and *MEK5*-targeting gRNA (5’-GAAGTGAATCTGAGACTGCT-3’) sequences were obtained from the Human CRISPR Knockout Pooled [50] and Bassik Lab Human CRISPR-*Cas9* Deletion [51] libraries, and inserted by Golden Gate cloning. Target BLM were lentivirally infected as described [52], reseeded, and after positive selection with 400 µg/ml Zeocin (InvivoGen, Toulouse, France) used polyclonally or picked and expanded as single cell clones.

### Generation of Cyclin D1/CDK4 overexpression cell lines and rescue experiments

For rescue experiments, Cyclin D1 and CDK4 overexpression constructs (pRc/CMV-Cyclin D1 and pCMV-HA-CDK4 [53]) or pCDNA3.1 empty vector (#V790-20, Invitrogen) were transfected into the respective BLM cell lines using Lipofectamine 3000 (#L3000008, ThermoFisher Scientific, Darmstadt, Germany), and stable polyclonal cell lines obtained by positive selection with 400 µg/ml G418 (Invivogen, Toulouse, France). For knockdown experiments, the respective naïve or Tram-pre-exposed cell lines were transfected with either scrambled siRNA 5’-UUCUCCGAACGUGUCACGUdTdT-3’ (Eurofins Genomics, Ebersberg, Germany) or siRNAs against *p21* (#SI00604905) or *p27* (#SI02621997) (Qiagen, Hilden, Germany) as described [7] and the following day treated with diluent, Tram or Tram/JWG-071 for 48 h.

### RNA isolation, cDNA synthesis and qRT-PCR

Total RNA isolation, cDNA synthesis and SYBR-green-based qRT-PCR using gene-specific qRT-PCR primer sequences (Supplementary Table 6) were performed as described previously [54] using an AriaMx cycler (Agilent, Waldbronn, Germany).

### RNA-seq and bioinformatic analysis

RNA-seq and bioinformatic analysis comparing differential gene expression between different groups were performed as detailed in the Supplementary Methods.

### Cell cycle profiling and BrdU labelling

Cell cycle profiling and BrdU labels were essentially performed as described [53]. Briefly, cells were fixed in 70% ethanol and stained with Propidium iodide (PI) alone or together with anti-BrdU-FITC antibody (BD Biosciences, Heidelberg, Germany). Subsequently, PI/anti-BrdU-FITC-stained cells were analyzed by flow-cytometry after doublet discrimination gating using the PI-A- and PI-W parameters. Further details are provided in the supplementary methods.

### Cell doubling-time analysis

BLM or BLM-based cells were seeded at a density of 5 × 10^3^ cells/cm^2^ and exposed the following day to vehicle- or 25 nM Tram. Subsequently, cells were incubated in the respective media, renewing media and inhibitors every 3-4 days. At ~80% confluency, cells were detached, counted and reseeded at the original density. After five weeks, experiments were terminated and cell doubling-times calculated and plotted against passage numbers as described [7].

## Supplementary information

Supplemental Material

Supp. Movie 1

Supp. Movie 2

Raw uncut western blots

## Data Availability

RNA-seq data are deposited in the GEO database (accession GSE300191). The materials generated during the current study are available upon request from the corresponding author.
