# Supplementary material for: MEK5/ERK5 inhibition sensitizes NRAS-mutant melanoma to MAPK-targeted therapy by preventing Cyclin D/CDK4-mediated G1/S progression
Source: Cell Death Dis. 2025 Oct 6;16(1):689. doi: 10.1038/s41419-025-08036-7 (PMC12501213; doi:10.1038/s41419-025-08036-7)
Supplement: Supplementary file 1 — Supplemental Material [file 41419_2025_8036_MOESM1_ESM.pdf]

SUPPLEMENTARY INFORMATION

MEK5/ERK5 inhibition sensitizes *NRAS*-mutant melanoma to MAPK-targeted therapy by preventing Cyclin D/CDK4-mediated G1/S progression

Rupesh Paudel, Simon Goller, Felix Deutzmann, Alina Gillitzer, Katharina Meder, Andrea Knorz, David Schrama, Matthias Goebeler, Marc Schmidt[#]

[#]Correspondence to: Prof. Dr. Marc Schmidt, Department of Dermatology, Venereology and Allergology, Josef-Schneider- Str. 2, 97080 Würzburg, Germany. E-mail: schmidt_M11@ukw.de, Phone: +49-931-201-26396

This file contains:

Supplementary Materials and Methods
Supplementary References
Supplementary Legends
- Figures
- Movies
- Tables

Supp. Materials and Methods

Time-lapse microscopy and crystal violet staining

The respective native or CRISPR/*Cas9*-modified BLM cell lines (BLM Wt, BLM *Scr* or BLM *MEK5*. k.o.) stably expressing empty vector (EV)- or Cyclin D1/CDK4 were pre-treated with vehicle or 25 nM Tram for two weeks and maintained in Tram- or Tram/JWG-071-containing medium for additional two weeks replacing media and inhibitors every 3-4 days. Subsequently, cells were re-seeded into 4- or 8-well Ibidi µ-Slides (IBIDI, Gräfeling, Germany) at a density of $2x10^4$ cells/cm^2 into the respective media. Once cells were attached (after ~3-4h), chamber slides were transferred to a Nikon Ti-E motorized stage fluorescence microscope equipped with a monochrome camera (Nikon, Düsseldorf, Germany) and a top stage incubator (Okulab, Naples, Italy) fitted with 5% CO_2 supply and maintained at 37°C. Time-lapse microscopy was performed by taking phase contrast photographs of each chamber with a speed of one frame/10 minutes over 72h using NIS-Elements AR 4.51.01 software (Nikon, Düsseldorf, Germany). Time-lapse photographs were assembled to movies, resized, stitched and provided with time-stamps using FIJI open-source imaging software (https://fiji.sc/). After time-lapse microscopy, chamber slides were either incubated for additionally three days or directly stained using home-made crystal violet solution (0.5% (w/v in methanol) as described previously (1).

RNA-seq and bioinformatic analysis

FM79 cells were seeded in triplicate and transfected with either scrambled siRNA or siRNA against *MEK5* (#SI00300713) (Qiagen, Hilden, Germany) as described previously (1). The following day, siRNA-transfected cells were either mock-treated or treated with 5 nM Tram for 48h prior to lysis and RNA extraction. RNA quality control (Bioanalyzer 2100, Agilent, Waldbrunn, Germany), cDNA library preparation (TruSeq Kit, Illumina, Cambridge, UK) and sequencing was performed at the Core Unit Systems Medicine of the University of Würzburg. Briefly, sequencing of pooled libraries, spiked with 1% PhiX control library, was performed at 38-47 million reads/sample in single-end mode with 75 nt read length on the NextSeq 500 platform (Illumina). Demultiplexed FASTQ files were generated with bcl2fastq v2.20.0.422 (Illumina).

To assure high sequence quality, Illumina reads were quality- and adapter-trimmed via Cutadapt (2) (v2.4) using a cutoff Phred score of 20 in NextSeq mode, and reads without any remaining bases were discarded (parameters: --nextseq-trim=20 -m 1 -a AGATCGGAAGAGCACACGTCTGAACTCCAGTCAC). Processed reads were subsequently mapped to the human genome (NCBI RefSeq assembly GCF_000001405.39/GRCh38.p13, primary assembly and mitochondrion) using STAR (3) v 2.7.1a with default parameters but

including transcript annotations from RefSeq annotation version 109.20190905 for GRCh38.p13. This annotation was also used to generate read counts on exon level summarized for each gene via featureCounts v1.6.4 from the Subread package (4). Multi-mapping and multi-overlapping reads were counted strand-specific and reversely stranded with a fractional count for each alignment and overlapping feature (parameters: -s 2 -t exon -M -O --fraction). The count output was utilized to identify differentially expressed genes using DESeq2 (5) v1.24.0. Read counts were normalized by DESeq2 and fold-change shrinkage was conducted by setting the parameter betaPrior to TRUE. Differentially expressed genes between different groups were compared in two arms: siScr vs Tram/SiScr =(Tram-regulated genes) or Tram/siScr vs Tram/si*MEK5* (=si*MEK5*-regulated genes) (Figure 1B) using DeSeq2. Only genes regulated above a threshold of ≥2.0 fold and an adjusted p-value <0.05 after Benjamini-Hochberg correction were considered as differentially expressed. Large scale promoter analysis using PSCAN 1.4 (6) as well as functional annotation cluster analysis using DAVID 6.8 (7) were essentially performed as described (8).

Cell cycle profiling

Cell cycle profiling was done by either Propidium iodide (PI) single staining or BrdU-incorporation assay coupled with PI staining. In both cases, the differently treated cells were harvested by trypsinization, pooling culture supernatants, PBS washing steps, and trypsinized cells into separate 15ml tubes for each condition to include any floating dead cells with subdiploid DNA content and detached mitotic cells in the analysis. For BrdU/PI-co-staining, cells were labelled by incubation with 1 µM of the base analog BrdU (Calbiochem #203806, Darmstadt, Germany) for 30 min prior to harvesting to allow incorporation of BrdU into actively replicating cells. Labelled or unlabelled cells were then pelleted by centrifuging at 240 g for 5 minutes followed by two washing steps with PBS. Finally, cells were fixed with 70% ice-cold ethanol and stored at 4 °C for at least overnight. For single PI staining, fixed cells subsequently were pelleted, and washed twice with PBS. Cells then were incubated with PI (1 µg/ml) supplemented with RNAse (250 µg/ml) for minimally one hour at room temperature in the dark to stain the DNA. Finally, DNA content of the was determined by flow-cytometry using a Cytoflex LX (BD, Biosciences, Heidelberg, Germany) flow cytometer analyzing PI-A fluorescence on a linear scale after non-doublet cell-gating based of the relations of the measured PI- W and PI- A parameters. Results were analyzed using FlowJo 10.10.0 Software (FlowJo LLC, Oregon, USA).

For BrdU/PI co-staining, ethanol-fixed cell pellets were treated with Pepsin (0.5 mg/ml in 0.1N HCl) for 20 minutes at room-temperature to obtain accessible cell nuclei. PBS/BSA/Tween-20 (0.1%/0.5%) solution was added and nuclei were centrifuged again before resuspending in 2N

HCl (12 minutes, at 37 °C). Borate buffer pH 8.5 was added to the suspension and nuclei pelleted again. Next, nuclei were washed with PBS/BSA/Tween-20 solution and stained with FITC mouse anti-BrdU antibody (BD # 347583, Heidelberg, Germany) at room temperature for one hour. Finally, nuclei were labelled with PI supplemented with RNAse as mentioned above and BrdU positivity determined by measuring logarithmic FITC-A and linear PI-A positively by flow cytometry using PI-A-PI-W parameter-based singlet gating.

Supp. References

1. Adam C, Fusi L, Weiss N, Goller SG, Meder K, Frings VG, et al. Efficient Suppression of NRAS-Driven Melanoma by Co-Inhibition of ERK1/2 and ERK5 MAPK Pathways. J Invest Dermatol. 2020;140(12):2455-65 e10.
2. Martin M. Cutadapt removes adapter sequences from high-throughput sequencing reads. EMBnetjournal. 2011;17(1):2.
3. Dobin A, Davis CA, Schlesinger F, Drenkow J, Zaleski C, Jha S, et al. STAR: ultrafast universal RNA-seq aligner. Bioinformatics. 2013;29(1):15-21.
4. Liao Y, Smyth GK, Shi W. featureCounts: an efficient general purpose program for assigning sequence reads to genomic features. Bioinformatics. 2014;30(7):923-30.
5. Love MI, Huber W, Anders S. Moderated estimation of fold change and dispersion for RNA-seq data with DESeq2. Genome Biol. 2014;15(12):550.
6. Zambelli F, Pesole G, Pavesi G. Pscan: finding over-represented transcription factor binding site motifs in sequences from co-regulated or co-expressed genes. Nucleic Acids Res. 2009;37(Web Server issue):W247-52.
7. Sherman BT, Hao M, Qiu J, Jiao X, Baseler MW, Lane HC, et al. DAVID: a web server for functional enrichment analysis and functional annotation of gene lists (2021 update). Nucleic Acids Res. 2022;50(W1):W216-W21.
8. Frings VG, Jopp L, Srivastava M, Presser D, Goebeler M, Schmidt M. Stress signalling and STAT1 activation characterize the keratinocytic gene expression pattern in Hidradenitis suppurativa. J Eur Acad Dermatol Venereol. 2022;36(12):2488-98.

Supp. legends

Supp. Figure 1: RNA-seq quality controls

A, B: mRNA expression data extracted from the RNA-seq dataset, validating efficient suppression of the ERK1/2 response gene *DUSP4* (**A**) and *MEK5* (**B**) by Tram and the employed *MEK5* siRNA, respectively. Bar diagrams show mean normalized fold reads per kilobase of transcript per million reads mapped (RPKM) + SD for the indicated groups relative to the unstimulated siScr control (arbitrarily set to 1), with data derived from n=3 independent experiments. Statistically significant differences between the indicated groups were evaluated by one-column *t*-test followed by Bonferroni multiplicity correction; ***p<0.001. **C:** Principal component analysis of the indicated groups for the three sequenced replicates. Corresponding samples of the respective independent experiments are marked with the same symbols, biological replicates from corresponding

treatment groups are highlighted by identical color and black circles. PCA analysis was generated using DeSeq2 (Bioconductor) (5). **D:** Volcano Plot, showing the top down- and upregulated genes (based on adjusted *p*-value) in Tram-treated, si*MEK5*-transfected FM79 cells versus Tram-treated, siScr-transfected FM79 **E:** Zoomed-in lower left corner of the downregulated genes containing multiple cell cycle relevant genes.

Supp. Figure 2: Kinetics of the MEKi/ERK5i-induced G1 arrest in melanoma cells

A: Time-dependent changes in DNA content, indicating rapid induction of a G1/S cell cycle arrest in response to concomitant Tram/XMD8-92 treatment of the specified treatment-naïve *NRAS*-mutant melanoma cell lines for the indicated times. Respective histograms show the results of flow cytometric DNA content analysis as determined by PI-mediated DNA staining. Calculated percentages of S-phase distribution for the respective samples are plotted above each histogram. *Top-left*: dot plot, illustrating the doublet exclusion strategy excluding the sub-G1 fraction using the 48h timepoint of Tram/XMD8-92-treated BLM as example. **B, C:** qPCR analysis of *CCND1* and *FOXM1* mRNA expression of M26 (**B**) or BLM cells (**C)** in response to MEKi/ERK5i co-exposure for eight to 24h. Tram concentrations used for M26 or BLM were 2.5 nM or 25 nM; XMD8-92 was used at 5 µM. Line diagrams represent mean fold GAPDH-normalized mRNA expression of the indicated genes + SD relative to the unstimulated 0h control with data derived from n=3 independent experiments. Statistically significant differences between the indicated timepoints and the respective 0h control group are indicated by asterisks (****p*<0.001, ***p*<0.01, **p*<0.05; one-column *t*-test with Bonferroni multiplicity correction). **D:** Representative immunoblots for the indicated EMT and differentiation markers in four different *NRAS*-mutant melanoma cells. Tubulin served as loading control.

Supp. Figure 3: MEKi/ERK5i inhibition reduces cellular proliferation of melanoma cells

Representative dot plots from n=2 experiments, showing flow cytometric analysis of BrdU positivity (BrdU-FITC, log scale) and DNA content (PI-A, linear scale) of the indicated NRAS-mutant melanoma cell lines with (FM79 and M26) or without (BLM and MaMel26a) basal ERK5 autophosphorylation activity after treatment for two days with Tram alone or Tram and the ERK5i XMD8-92. Cells were pulsed with BrdU for 30 min at the end of the incubation time and percentages of BrdU incorporation, indicating replicating cells, were determined by combined αBrdU-FITC and PI co-staining. Quantification was performed after PI-W/ PI-A non-doublet gating. Numbers in the upper left corner of the respective dot blots denote the percentage of replicating cells present in the indicated quantification gate.

Supp. Figure 4: ERK5i co-administration induces a rapid G1 arrest in Tram^R melanoma cells

A: Gradual acquisition of Tram resistance (Tram^R) by *NRAS*-mutant BLM melanoma cells after continuous culture in 25 nM Tram-containing medium. Histograms show DNA profiles obtained by flow cytometric analysis of PI-stained cells harvested after the indicated time periods (days, d) of Tram treatment. For each DNA profile, calculated proportions of S-phase cells are indicated. **B:** Immunoblots from single long-term experiment for EMT and differentiation marker proteins in total lysates of BLM cells treated with Tram for the one day to four weeks. Tubulin served as loading control. **C, D:** Line diagrams, illustrating a rapid decline in the percentage of S-phase cells with concomitant accumulation of G1 cell fractions following co-treatment of Tram-resistant BLM (BLM Tram^R) with 5 µM of the ERK5i JWG-071 (**C**) or XMD8-92 (**D**). Shown are mean percentages of S-phase or G1 cells + SD for the indicated timepoints from n=3 independent experiments. For all biological replicates, Tram^R cells were independently generated by 14 days of Tram pretreatment and re-seeded at equal densities into Tram-containing medium prior to stimulation with diluent (0h time-points) or the two ERK5i. Statistical differences of the ERK5i-stimulated samples in (**C**) and (**D**) and the common Tram mono-stimulated 0h control was evaluated by one-way ANOVA with post-Dunnet test; (***p<0.001, **p<0.01, *p<0.05). **E:** *CCND1*, *FOXM1* and *CCNE2* mRNA expression of Tram^R BLM prepared as in (**C, D**), following exposure to Tram alone or Tram together with 5 µM XMD8-92 or JWG-071 for 8h. Data represent mean (n=3) fold *GAPDH*-normalized mRNA expression + SD relative to the Tram-monotreated control (set to 1). Colored lines illustrate the treatment scheme. Statistical difference of the indicated groups in relation to the monotreated Tram control is indicated by asterisks (**p<0.01, *p<0.05; one-column *t*-test with Bonferroni multiplicity correction).

Supp. Figure 5: Inability of ectopic Cyclin D1/CDK4 to completely reverse G1 arrest induced by concurrent Tram/ERK5i incubation of drug-naïve BLM or short-term ERK5i addition to Tram^R BLM

A: Quantification S-phase distribution of Tram^R BLM stably expressing an empty vector (EV) or Cyclin D1/CDK4 following Tram/JWG-071 co-treatment for two days. Values shown as mean + SD percentile from n=3 experiments, in which Tram resistance was induced by >2 weeks pre-incubation in a Tram-containing medium. (ns: p>0.05, two-way ANOVA with post-Sidak test) **B:** Representative immunoblots n=3 for the indicated cell cycle proteins using total lysates from empty vector (EV)- or stably Cyclin D1/CDK4-expressing BLM treated with Tram or Tram/JWG-071 for 14 days (d). **C:** Flow-cytometric DNA profiling analysis of a representative experiment out of n=3 performed in parallel to **(B)**. Values denote percentages of S-phase cells for the respective

condition. **D:** Quantification of percent S-phase distribution of experiments done in parallel to **(B)** (ns: *p*>0.05, two-way ANOVA with post-Sidak test). **E:** Quantification of the percentage S-phase distribution of Tram^R BLM stably expressing an empty vector (EV) or Cyclin D1/CDK4 following transfection with the indicated siRNA and subsequent Tram/JWG-071 co-treatment for two days. Values shown as mean + SD percentile S-phase distribution from n=3 experiments, in which Tram resistance was induced by >2 weeks pre-incubation in a Tram-containing medium. Asterisks indicate statistical differences between the specified condition and the respective siScr control (****p*<0.001, ***p*<0.01, **p*<0.05, two-way ANOVA with post-Sidak test). Colored lines in **(D)** and (**E**) illustrate the treatment schemes.

Supp. Figure 6: Forced Cyclin D1/CDK4 expression prevents MEKi-induced G1 arrest in *MEK5*-deficient cells and reverses the anti-proliferative effects of sustained MEKi/ERK5i exposure in Tram^R melanoma cells

A: S-phase distribution (**top**) or corresponding crystal violet staining (**bottom**) of n=3 empty vector (EV)- or stably Cyclin D1/CDK4-expressing BLM after acquisition of Tram resistance (Tram^R) by two weeks of Tram-pretreatment and subsequent Tram/JWG-071 co-treatment for an additional 14 (**top**) or 20 days (**bottom**), respectively. For comparison, results from treatment-naïve BLM with corresponding genetic manipulations are shown. Cells were reseeded at equal density one (**top**) or six days (**bottom**) prior to analysis. Asterisks indicate statistical differences between the two indicated groups (****p*<0.001, two-way ANOVA with post-Sidak test). **B:** Representative cell cycle profiles of n=3 for BLM, Scr-infected BLM, or CRISPR/*Cas9*-mediated *MEK5* k.o cells, expressing either EV- or stably Cyclin D1/CDK4 after treatment with diluent (ctrl) or Tram for 14 days. **C**: Quantification of S-phase distribution from n=3 experiments conducted as in **B** (**top**) and corresponding crystal violet staining (**bottom**) after 17 days of Tram treatment (**bottom**). Bar diagrams represent mean percentage S-phase distribution + SD of n=3 independent experiments. Asterisks indicate statistical differences between the indicated groups (****p*<0.001, ***p*<0.01, two-way ANOVA with post-Sidak test). The crystal violet staining shows a corresponding representative experiment out of n=3. Colored lines indicate the respective treatment schemes.

Supp. Movie 1: Time-lapse microscopy, visualizing the effect of ectopic Cyclin D1/CDK4 expression on MEKi-resistant cells exposed to sustained MEKi/ERK5i

Empty vector (EV)- or stably Cyclin D1/CDK4-expressing BLM were left untreated (ctrl) or Tram pre-treated for two weeks to induce Tram-resistance (Tram^R) and then further incubated with Tram alone or Tram in combination with the ERK5i JWG-071 for an additional two weeks. The treated conditions were then reseeded at equal density into 8-well µ-slides and cell proliferation was followed over a period of 72h by multi-position phase contrast time-lapse microscopy.

Representative movies of n=3 independent experiments recorded at one frame every ten minutes are shown. Scale bar = 100 µM. (Top right: EV + control (ctrl), Top middle: EV + Tram, Top left: EV + Tram/JWG-071(JWG), Bottom right: Cyclin D1/CDK4 + ctrl, Bottom middle: Cyclin D1/CDK4 + Tram, Bottom left: Cyclin D1/CDK4+Tram/JWG-071 (JWG)).

Supp. Movie 2: Time-lapse microscopy, demonstrating reversion of MEKi-induced cell cycle arrest of MEK5-deficient melanoma cells upon CyclinD1/CDK4 expression

Scrambled (Scr)-infected BLM, or CRISPR/*Cas9*-mediated *MEK5* k.o. BLM, stably expressing either empty vector (EV) or Cyclin D1/CDK4, were treated with diluent (control) or Tram for 14 days. All four Tram-treated conditions were then reseeded at equal density into 4-well µ-slides and proliferation was monitored over a period of 72h by multi-position phase contrast time-lapse microscopy. Representative movies of n=3 recorded at one frame/10 min are shown. Scale bar = 100 µM. (Top right: Scr Control (Scr) + EV, Top left: *MEK5* k.o. + EV, Bottom right: Scr Control (Scr) + Cyclin D1/CDK4, Bottom left: *MEK5* k.o. + Cyclin D1/CDK4).

Supp. Table 1: List of statistically ≥2.0-fold si*MEK5*- dependently downregulated genes present in the overrepresented functional annotation cluster "nuclear division"

Supp. Table 2: List of selected Tram and Tram+*siMEK5* co-regulated G1S genes

Supp. Table 3: List of overrepresented functional annotation terms obtained by functional annotation cluster analysis of ≥2.0-fold Tram- and *siMEK5* co-downregulated DEGs

DAVID functional annotation cluster analysis of the statistically (p<0.05) *siMEK5* and Tram co-downregulated DEGs. Functional clusters are ranked by the mean enrichment score (negative logarithm of mean collective *p*-values) of the gene ontology biological process functional annotation terms included in the respective cluster.

Supp. Table 4: List of overrepresented transcription factor as identified by large scale promoter analysis performed on the ≥2.0-fold Tram and *siMEK5* co-downregulated DEGs

Supp. Table 5: List of antibodies used

Supp. Table 6: List of qRT-PCR primer used

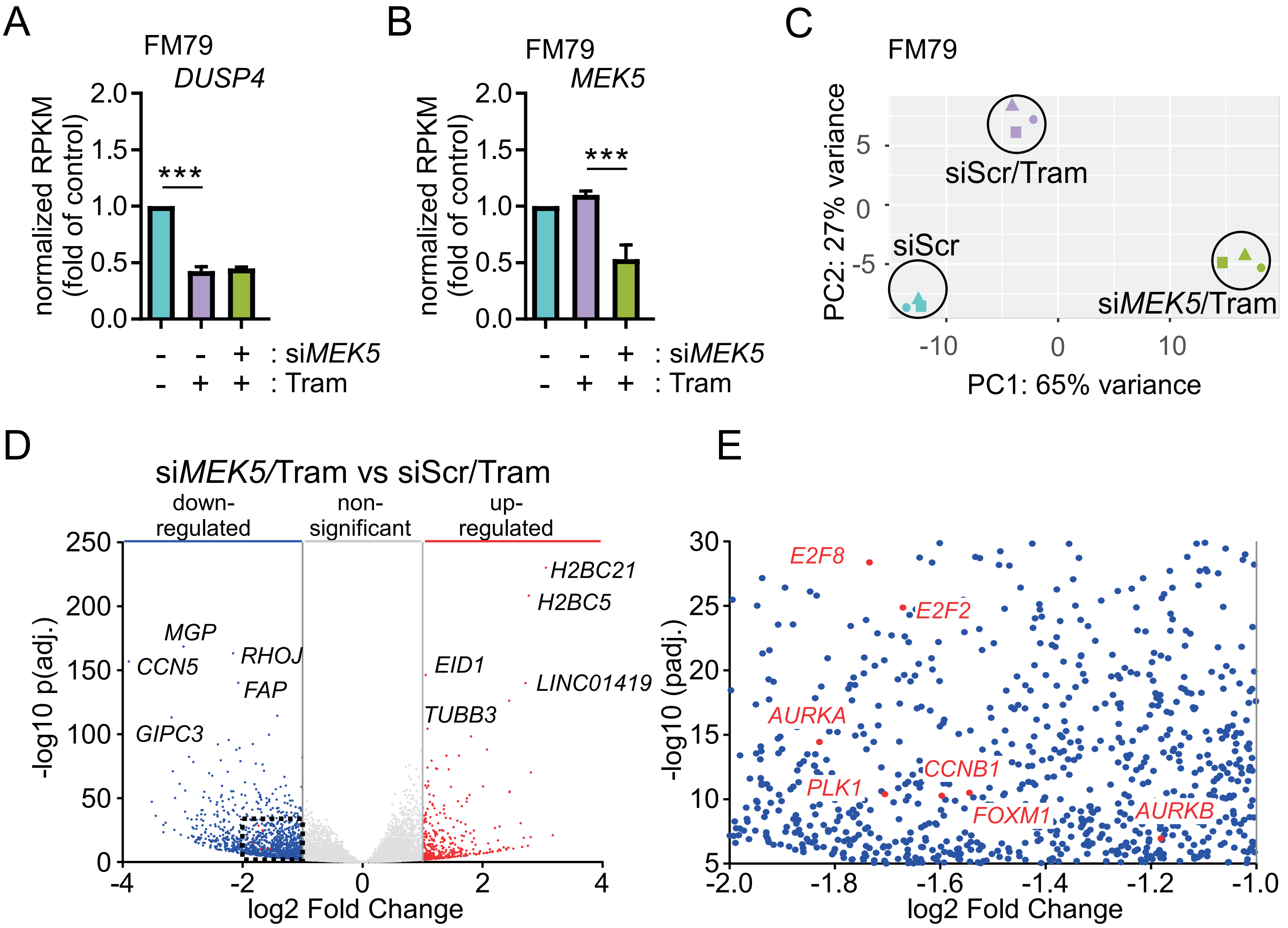

Supp. Figure 1

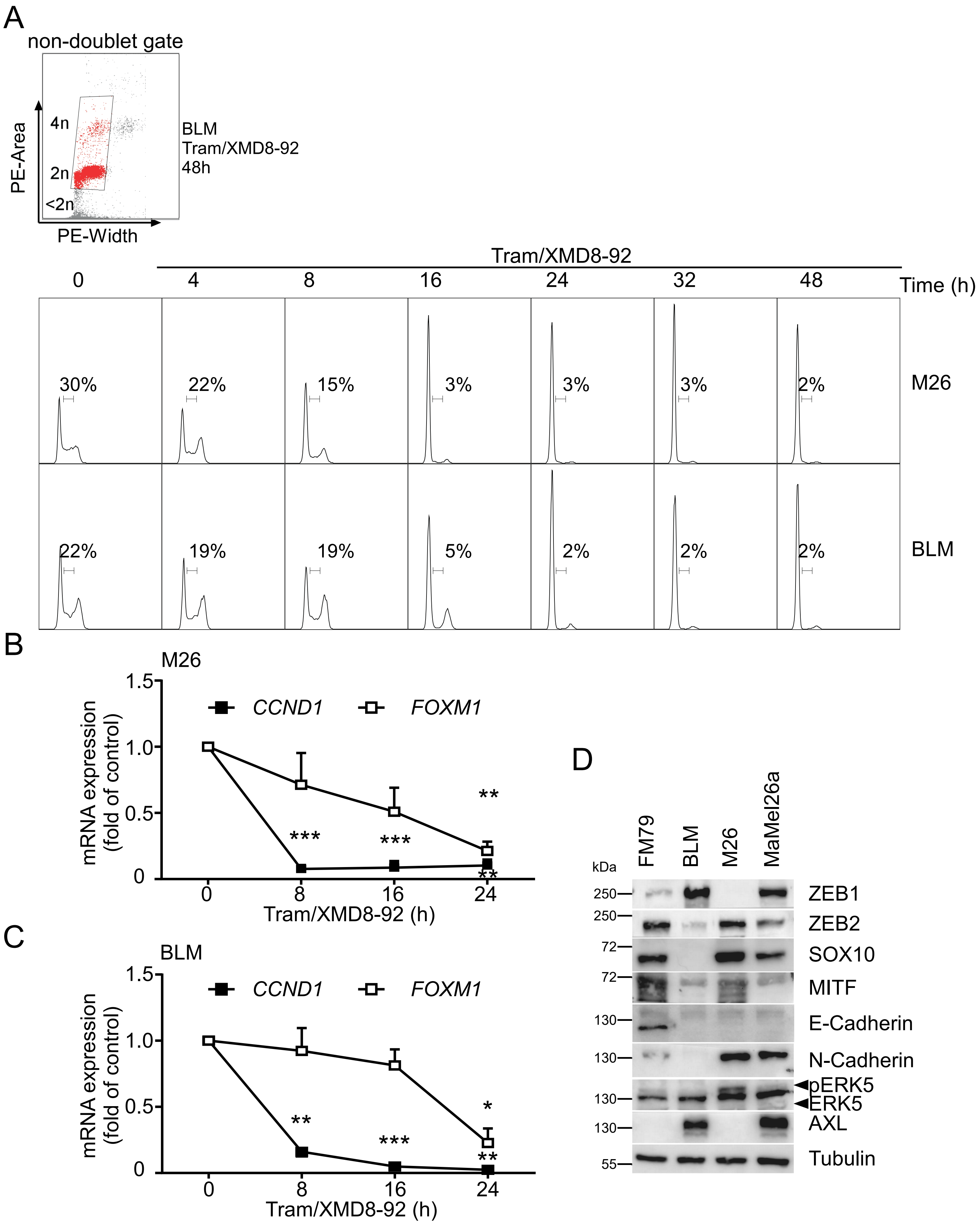

Supp. Figure 2

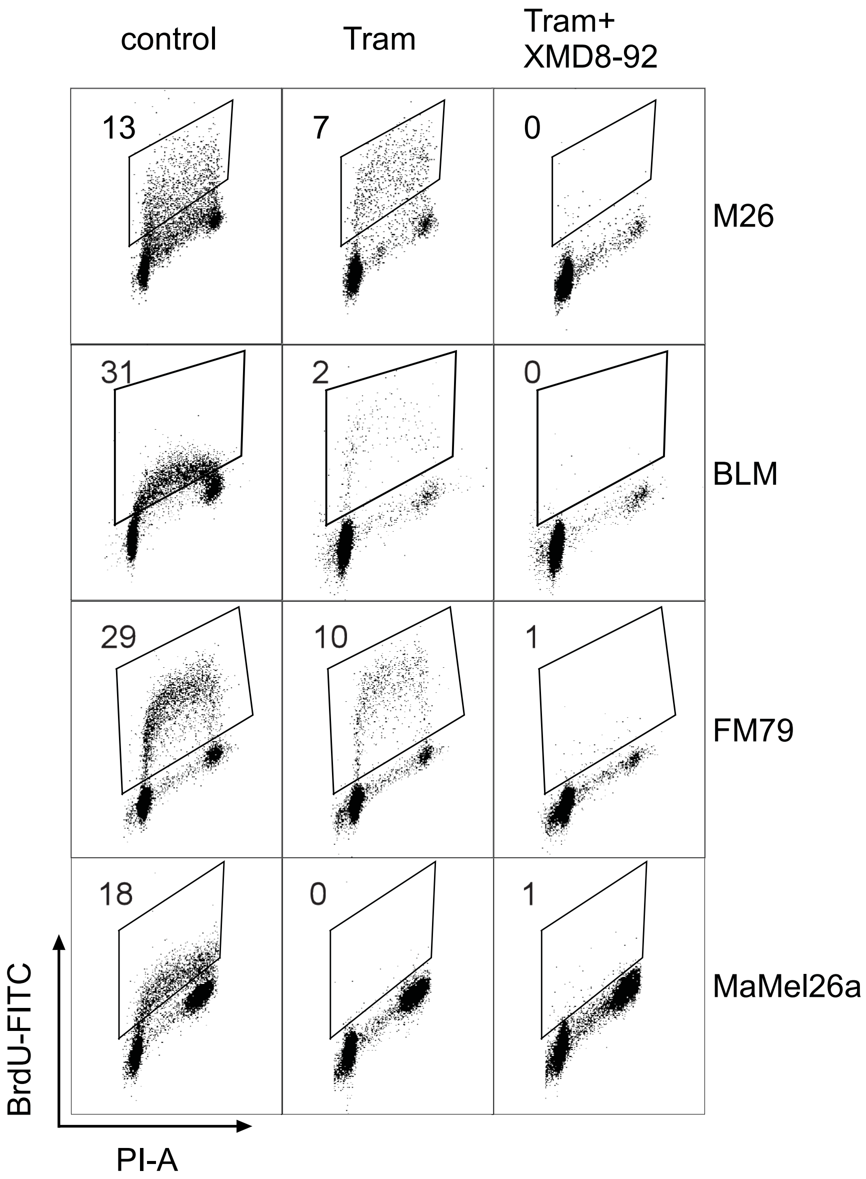

Supp. Figure 3

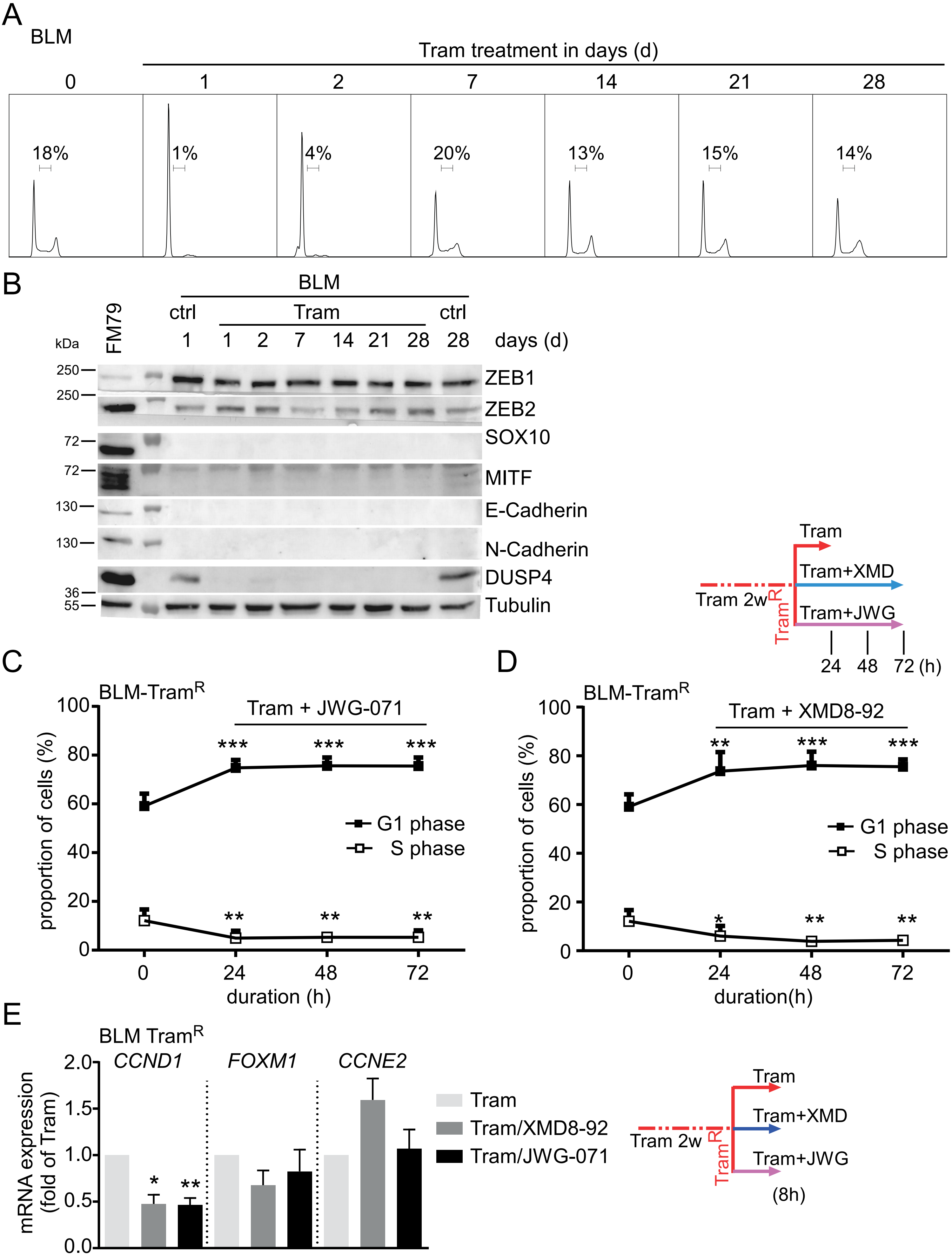

Supp. Figure 4

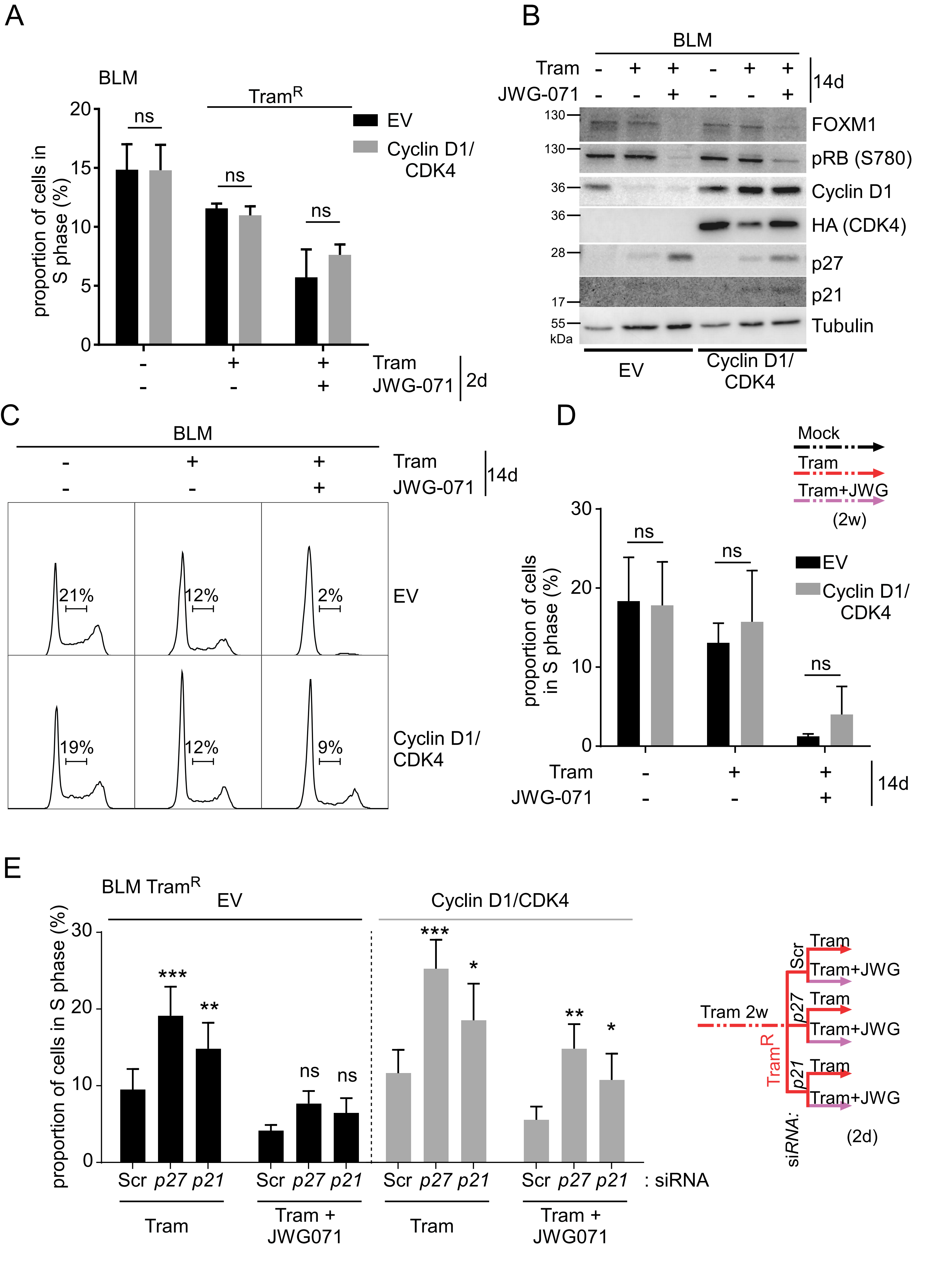

Supp. Figure 5

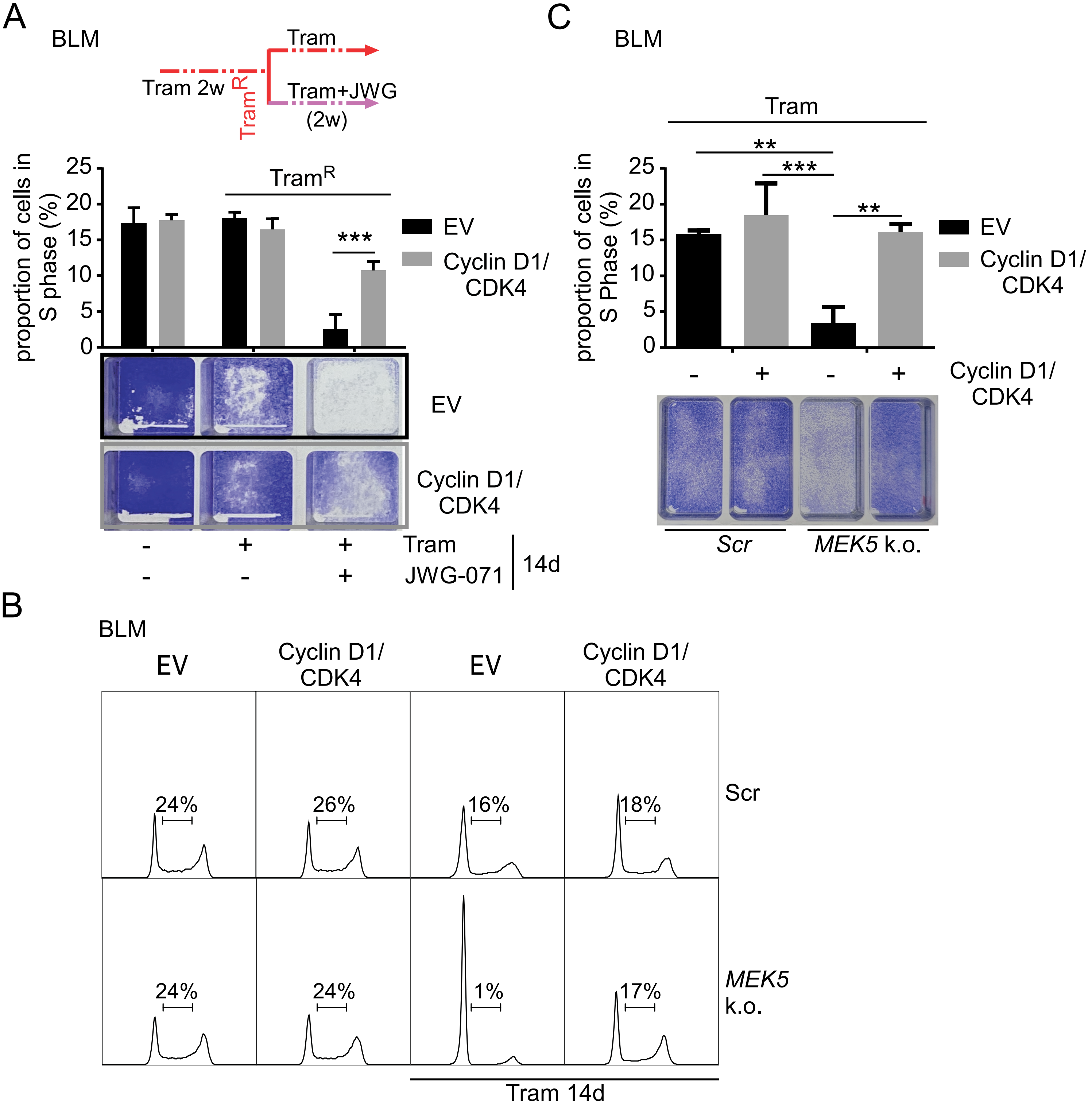

Supp. Figure 6

Supp. Table 1

serial number	Gene	Gene full name	fold repression by MEK5 siRNA
1	*LIF*	leukemia inhibitory factor(LIF)	8,960293929
2	*CYP26B1*	cytochrome P450 family 26 subfamily B member 1(CYP26B1)	4,692829038
3	*IL1B*	interleukin 1 beta(IL1B)	4,055820799
4	*IGF1*	insulin like growth factor 1(IGF1)	3,886283893
5	*PDGFB*	platelet derived growth factor subunit B(PDGFB)	3,575834823
6	*MEIOC*	meiosis specific with coiled-coil domain(MEIOC)	3,573357563
7	*EGF*	epidermal growth factor(EGF)	3,566646071
8	*AURKA*	aurora kinase A(AURKA)	3,553660036
9	*BIRC3*	baculoviral IAP repeat containing 3(BIRC3)	3,442405466
10	*ANKLE1*	ankyrin repeat and LEM domain containing 1(ANKLE1)	3,352834028
11	*PLK1*	polo like kinase 1(PLK1)	3,260942248
12	*BIRC7*	baculoviral IAP repeat containing 7(BIRC7)	3,248159898
13	*CDCA3*	cell division cycle associated 3(CDCA3)	3,203996919
14	*KIF18B*	kinesin family member 18B(KIF18B)	3,128971927
15	*CDC25C*	cell division cycle 25C(CDC25C)	3,125350817
16	*NUF2*	NUF2, NDC80 kinetochore complex component(NUF2)	3,115227725
17	*PBK*	PDZ binding kinase(PBK)	3,113853168
18	*MEIOB*	meiosis specific with OB domains(MEIOB)	3,06414916
19	*FSD1*	fibronectin type III and SPRY domain containing 1(FSD1)	3,034255356
20	*CCNB1*	cyclin B1(CCNB1)	3,025362661
21	*KIF14*	kinesin family member 14(KIF14)	3,017344111
22	*ESPL1*	extra spindle pole bodies like 1, separase(ESPL1)	2,936812847
23	*pkmyt1*	protein kinase, membrane associated tyrosine/threonine 1(PKMYT1)	2,869127487
24	*TOP2A*	topoisomerase (DNA) II alpha(TOP2A)	2,84741129
25	*MKI67*	marker of proliferation Ki-67(MKI67)	2,833661591
26	*KIF4B*	kinesin family member 4B(KIF4B)	2,832934078
27	*SGO1*	shugoshin 1(SGO1)	2,789831168
28	*CCNA2*	cyclin A2(CCNA2)	2,78573523
29	*ASPM*	abnormal spindle microtubule assembly(ASPM)	2,776395926
30	*DLGAP5*	DLG associated protein 5(DLGAP5)	2,763305867

31	*PRC1*	protein regulator of cytokinesis 1(PRC1)	2,75451293
32	*CEP55*	centrosomal protein 55(CEP55)	2,735672682
33	*KIFC1*	kinesin family member C1(KIFC1)	2,727175457
34	*NDC80*	NDC80, kinetochore complex component(NDC80)	2,726736177
35	*TPX2*	TPX2, microtubule nucleation factor(TPX2)	2,720620448
36	*CDCA8*	cell division cycle associated 8(CDCA8)	2,679611266
37	*KIF4A*	kinesin family member 4A(KIF4A)	2,660637887
38	*NEK2*	NIMA related kinase 2(NEK2)	2,65719944
39	*MYBL2*	MYB proto-oncogene like 2(MYBL2)	2,638379649
40	*KIF2C*	kinesin family member 2C(KIF2C)	2,611841708
41	*SPC25*	SPC25, NDC80 kinetochore complex component(SPC25)	2,58895819
42	*STAG3*	stromal antigen 3(STAG3)	2,588327358
43	*FAM83D*	family with sequence similarity 83 member D(FAM83D)	2,582924966
44	*CENPA*	centromere protein A(CENPA)	2,568658137
45	*PSRC1*	proline and serine rich coiled-coil 1(PSRC1)	2,55910034
46	*KIF23*	kinesin family member 23(KIF23)	2,558546742
47	*CDC20*	cell division cycle 20(CDC20)	2,551853072
48	*RACGAP1*	Rac GTPase activating protein 1(RACGAP1)	2,519798824
49	*SPAG5*	sperm associated antigen 5(SPAG5)	2,518223361
50	*BIRC5*	baculoviral IAP repeat containing 5(BIRC5)	2,498752792
51	*USP44*	ubiquitin specific peptidase 44(USP44)	2,486442681
52	*CDCA2*	cell division cycle associated 2(CDCA2)	2,479433093
53	*SPC24*	SPC24, NDC80 kinetochore complex component(SPC24)	2,47335835
54	*CDC25A*	cell division cycle 25A(CDC25A)	2,472072631
55	*BUB1B*	BUB1 mitotic checkpoint serine/threonine kinase B(BUB1B)	2,433962201
56	*MSX1*	msh homeobox 1(MSX1)	2,417048282
57	*CCNA1*	cyclin A1(CCNA1)	2,400592539
58	*FAM64A*	family with sequence similarity 64 member A(FAM64A)	2,399833201
59	*PDE3A*	phosphodiesterase 3A(PDE3A)	2,397246267
60	*KIF22*	kinesin family member 22(KIF22)	2,391751114
61	*UBE2C*	ubiquitin conjugating enzyme E2 C(UBE2C)	2,373919536
62	*NCAPG*	non-SMC condensin I complex subunit G(NCAPG)	2,366411491
63	*CDCA5*	cell division cycle associated 5(CDCA5)	2,361171292

64	*CCNB2*	cyclin B2(CCNB2)	2,350796986
65	*BUB1B-PAK6*	BUB1B-PAK6 readthrough(BUB1B-PAK6)	2,346720663
66	*SKA3*	spindle and kinetochore associated complex subunit 3(SKA3)	2,331505694
67	*BORA*	bora, aurora kinase A activator(BORA)	2,317891957
68	*TRIP13*	thyroid hormone receptor interactor 13(TRIP13)	2,300348503
69	*TTK*	TTK protein kinase(TTK)	2,298387673
70	*DMC1*	DNA meiotic recombinase 1(DMC1)	2,288783442
71	*P3H4*	prolyl 3-hydroxylase family member 4 (non-enzymatic)(P3H4)	2,288198002
72	*CENPE*	centromere protein E(CENPE)	2,281063095
73	*SMC2*	structural maintenance of chromosomes 2(SMC2)	2,276366626
74	*OIP5*	Opa interacting protein 5(OIP5)	2,269942413
75	*NUSAP1*	nucleolar and spindle associated protein 1(NUSAP1)	2,269015467
76	*AURKB*	aurora kinase B(AURKB)	2,267719449
77	*BMP7*	bone morphogenetic protein 7(BMP7)	2,254604615
78	*PTTG1*	pituitary tumor-transforming 1(PTTG1)	2,249484034
79	*FBXO43*	F-box protein 43(FBXO43)	2,238544265
80	*KIF11*	kinesin family member 11(KIF11)	2,232988236
81	*PDGFRB*	platelet derived growth factor receptor beta(PDGFRB)	2,220368322
82	*LRRCC1*	leucine rich repeat and coiled-coil centrosomal protein 1(LRRCC1)	2,184887504
83	*CENPF*	centromere protein F(CENPF)	2,174330484
84	*ANLN*	anillin actin binding protein(ANLN)	2,160815885
85	*SGO2*	shugoshin 2(SGO2)	2,132544509
86	*CAV2*	caveolin 2(CAV2)	2,125364637
87	*RAD54L*	RAD54-like (S. cerevisiae)(RAD54L)	2,121303981
88	*DAZL*	deleted in azoospermia like(DAZL)	2,101243026
89	*SYCP2*	synaptonemal complex protein 2(SYCP2)	2,088568611
90	*CDK1*	cyclin dependent kinase 1(CDK1)	2,076004056
91	*KIF15*	kinesin family member 15(KIF15)	2,067993005
92	*ercc6l*	ERCC excision repair 6 like, spindle assembly checkpoint helicase(ERCC6L)	2,041119988
93	*MIS18BP1*	MIS18 binding protein 1(MIS18BP1)	2,041034061
94	*CIT*	citron rho-interacting serine/threonine kinase(CIT)	2,014138503
95	*RAD51*	RAD51 recombinase(RAD51)	2,012943772
96	*TACC3*	transforming acidic coiled-coil containing protein 3(TACC3)	2,004440302

Supp. Table 2

serial number	Gene description	Gene	Tram-regulated (Fold change)	siMEK5-regulated (Fold change)	Total fold change	Fold suppression
1	cyclin A1	*CCNA1*	0,4016	0,4166	0,1673	-5,9780
2	cyclin A2	*CCNA2*	0,7565	0,3590	0,2715	-3,6826
3	cyclin dependent kinase inhibitor 1C (p57 KIP2)	*CDKN1C*	0,5664	0,4984	0,2823	-3,5426
4	cyclin dependent kinase inhibitor 2C (p18)	*CDKN2C*	0,9153	0,3438	0,3146	-3,1783
5	cyclin D1	*CCND1*	0,5382	0,6594	0,3549	-2,8177
6	cyclin E2	*CCNE2*	0,6849	0,5577	0,3820	-2,6178
7	F-box protein 5 (EMI1)	*FBXO5*	0,7243	0,5615	0,4067	-2,4587
8	cyclin dependent kinase inhibitor 1A (p21 Cip1)	*CDKN1A*	0,9948	0,6684	0,6649	-1,5039
9	cyclin E1	*CCNE1*	0,8216	0,8216	0,6750	-1,4815
10	MYC proto-oncogene%2C bHLH transcription factor	*MYC*	0,6426	1,0831	0,6960	-1,4369
11	cyclin D3	*CCND3*	0,8989	0,7986	0,7179	-1,3930
12	cyclin dependent kinase inhibitor 2A (p14/p16/p19)	*CDKN2A*	0,8357	0,8846	0,7392	-1,3528
13	cyclin dependent kinase inhibitor 1B (p27 KIP1)	*CDKN1B*	1,2135	0,7500	0,9100	-1,0988
14	cyclin dependent kinase inhibitor 2B (p15)	*CDKN2B*	1,1070	1,2605	1,3954	-0,7167

Supp. Table 3

serial number	functional annotation terms	Enrichment Score (-log (pValue))	Genes involved in cluster
1	mitotic cell cycle phase transition	18,1	CDKN1C, CCNF, KIF14, BUB1B, BRCA1, CKS1B, MTBP, CCND1 , CHEK2, DPF1, NUF2, CHEK1, KNTC1, FBXO5 , SPDL1, POLE, CDK5RAP2, CTDSPL, KNL1, HASPIN, MASTL, CDC25C, CDC25A, CIT, CCNA1, DBF4, MELK, CCNE2, ESPL1, CKS2, DTL, BLM, RNASEH2B, CDCA5, PKMYT1, AURKB, AURKA, CCNB1, ORC1, TERT, E2F2, CLSPN, BUB1, CDKN2D, BARD1, STIL, RRM1, RRM2, XRCC3, HMGA2, INHBA, ZWINT, TICRR, BUB1B-PAK6, CENPE, CDK6, UBE2S, CENPJ, CYP1A1, SASS6, TACC3, SPC24, SPC25, EZH2, MAD2L1
2	regulation of chromosome segregation	16,9	FEN1, CDCA2, CDCA5, NCAPG2, NCAPG, BUB1B, SMC4, NCAPH, AURKB, SMC2, CCNB1, PTTG1, DPF1, NUF2, KNTC1, FBXO5 , SPDL1, BUB1, CDK5RAP2, RMI2, SPAG5, XRCC3, KNL1, HASPIN, ZWINT, BUB1B-PAK6, CENPE, PLSCR1, ESPL1, TACC3, KIF2C, NCAPD2, SPC24, MAD2L1, SPC25
3	meiotic cell cycle	13,9	TOP2A, ANKLE1, SYCP2, BUB1B, PKMYT1, FBXO43, BRCA2, SMC4, NCAPH, AURKA, SMC2, PSMC3IP, RAD51AP1, BRIP1, CYP26B1, PTTG1, EXO1, WBP2NL, NUF2, MEIOB, RAD54L, FBXO5 , BUB1, NDC1, FAM9C, XRCC2, LIF, FANCA, MND1, TEX15, MASTL, SGO1, CCNA1, SGO2, ESPL1, STAG3, CCNE2, FANCD2, CKS2, NCAPD2, MNS1
4	negative regulation of cell cycle process	11,7	BLM, CCNF, BUB1B, BRCA1, FBXO43, AURKB, MTBP, CCNB1, ORC1, PTTG1, CCND1 , CHEK2, NUF2, CHEK1, KNTC1, CLSPN, FBXO5 , SPDL1, BUB1, CDK5RAP2, E2F8, BARD1, CDKN2D, RBM14, XRCC3, DAB2IP, LIF, CTDSPL, HMGA2, INHBA, KNL1, HASPIN, ZWINT, TICRR, BUB1B-PAK6, ESPL1, DTL, SPC24, EZH2, MAD2L1, SPC25
5	regulation of chromosome separation	11,2	NCAPG2, NCAPG, BUB1B, SMC4, NCAPH, AURKB, SMC2, CCNB1, PTTG1, DPF1, NUF2, KNTC1, FBXO5 , SPDL1, BUB1, CDK5RAP2, XRCC3, KNL1, HASPIN, ZWINT, BUB1B-PAK6, CENPE, PLSCR1, ESPL1, TACC3, NCAPD2, SPC24, MAD2L1, SPC25
6	centrosome organization	10,5	CCNF, BRCA1, WDR62, KIF11, BRCA2, AURKA, HAUS8, HAUS7, CHEK1, PCLAF, CEP72, POC1B, CDK5RAP2, PLK4, RANBP1, RBM14, STIL, CEP152, XRCC2, XRCC3, HAUS1, SGO1, CENPJ, SASS6, SSX2IP
7	cell cycle G2/M phase transition	7,9	BLM, RNASEH2B, KIF14, BRCA1, PKMYT1, AURKB, AURKA, CCNB1, ORC1, CCND1 , CHEK2, CHEK1, CLSPN, FBXO5 , BARD1, RRM1, ATAD5, HMGA2, MASTL, CDC25C, CDC25A, TICRR, CIT, MELK, CDK6, DTL
8	reproduction	7,7	ANKLE1, SERPINE2, CCNF, TNC, BUB1B, SMC4, PLB1, SMC2, PTTG1, CCND1 , MEIOB, FBXO5 , PHLDA2, SLC38A1, IL4R, IGFBP5, KNL1, CDC25C, ADAM19, SGO1, SGO2, CCNE2, FANCD2, OSBP2, PKMYT1, FBXO43, HIF1A, CYP19A1, NCAPH, RAD51AP1, C3, CYP27B1, BRIP1, WBP2NL, B4GALNT1, SLC19A1, PLK4, TFAP2C, FAM9C, VDR, XRCC2, FANCA, LIF, INHBA, FANCG, STAG3, HOOK1, MAFF, CYP1A1, SNAI1, HROB, CDKN1C, TOP2A, SYCP2, MCM8, ARRB2, BRCA2, SERPINA5, CYP26B1, EXO1, NUF2, TIMP1, NDC1, MME, TESC, EMP2, FOS, TEX15, MASTL, CCNA1, IL1A, ESPL1, IL1B, CKS2, MNS1, BIRC3, CEBPB, LEF1, ADCY3, HOXD13, LIN9, ADCY7, AURKA, PSMC3IP, RAD54L, HES1, BUB1, E2F8, NTRK1, RRM1, MND1, TBX3, CTCFL, VEGFA, SPAG1, PTX3, NCAPD2
9	apoptotic process	6,7	STEAP3, ITPRIP, BUB1B, ICAM1, IL27RA, CCND1 , RASSF5, CHEK2, DPF1, CHEK1, MYB, PDK4, ADORA1, TNFRSF8, CHAC1, PHLDA2, MAP3K5, TGM2, PRKCH, TRAIP, OLFM1, MAG, MELK, LGALS12, TRIB3, EPHA2, CSF1R, USP53, CDCA7, HIF1A, CORO1A, TERT, BARD1, STIL, EGR3, DDIAS, TNFSF15, VDR, XRCC2, TNFRSF10B, HMGA2, TNFRSF10A, INHBA, NET1, NR4A1, CD28, SNAI1, TP73, TOP2A, NRP1, DRAXIN, SYCP2, MEGF10, KIF14, TNFAIP3, ARRB2, BRCA1, BRCA2, CTSS, TIMP3, CTLA4, TIMP1, SRGN, HELLS, SPHK1, CKAP2, AEN, EMP2, TRAF1, TENT5B, TNFRSF1B, CIT, ALDH1A3, IL1A, PLSCR1, AIM2, ESPL1, IRF1, IL1B, MIR17HG, ARHGEF2, ITGB3BP, BIRC3, MCM2, COLEC11, CEBPB, MTFP1, LEF1, AMIGO2, MALT1, AURKB, AURKA, FRZB, E2F2, BUB1, CDKN2D, NTRK1, CD74, ATAD5, DAB2IP, LAPTM5, TBX3, VEGFA, KRT18, HDGF, EVI2B, CRLF1, MAD2L1
10	centriole replication	6,5	PLK4, RBM14, STIL, CEP152, CENPJ, SASS6, CEP72, POC1B, BRCA1, WDR62, CDK5RAP2
11	cell cycle DNA replication initiation	5,3	POLA1, GINS3, MCM3, MCM4, MCM2
12	metaphase plate congression	4,8	CDCA5, KIF14, KIF22, GEM, AURKB, CENPE, CCNB1, PSRC1, NUF2, KIF2C, SPDL1, FAM83D, CENPQ
13	regulation of cell activation	4,6	SERPINE2, TNFAIP3, PTPN22, IL27RA, MILR1, ADGRE2, CYP26B1, C1QTNF1, UBASH3B, RASSF5, MYB, RAC2, CTLA4, EPHB2, HLA-DOA, IL4R, SPHK1, TNFRSF1B, IL1A, PLSCR1, CEACAM1, FANCD2, ADORA2B, IRF1, IL1B, MIR17HG, HLA-DQB1, CEBPB, TCF7, LEF1, CORO1A, MALT1, FCHO1, HLX, NSD2, SLAMF8, HES1, SH2B3, CD74, EGR3, ATAD5, LAPTM5, FANCA, INHBA, NR1D1, NR4A3, CD28, HLA-DRA, CD22, CD200
14	deoxyribose phosphate biosynthetic process	4,4	RRM1, DTYMK, RRM2, SHMT1, CMPK2, AK5, TYMS
15	regulation of protein modification process	4,3	CCNF, BUB1B, ICAM1, MTBP, CCND1 , UBASH3B, RASSF5, CHEK2, CHEK1, MYB, ADORA1, FBXO5 , EPHB2, MAP3K5, CDK5RAP2, BORA, CDC25C, CDC25A, CCNE2, TRIB3, TRABD2A, EPHA2, CSF1R, BLM, CDCA2, PKMYT1, HIF1A, C3, CCNB1, CLSPN, FAM83D, FANCI, CCR1, TNFSF15, LIF, TNFRSF10B, HMGA2, TNFRSF10A, INHBA, RCAN1, UBE2S, CCDC88C, CSPG4, CHRFAM7A, PHF19, EZH2, TP73, CDKN1C, NRP1, SUV39H1, KIF14, TNFAIP3, PTPN22, ARRB1, ARRB2, BRCA1, C1QTNF1, PBK, PMEPA1, TIMP3, IL11, SPHK1, CTDSPL, EMP2, TRAF1, MASTL, CCNA1, IL1A, CEACAM1, PSRC1, IL1B, ARHGEF2, BIRC3, AVPI1, OPRD1, RCHY1, CAMKK1, MALT1, AURKB, HES1, SH2B3, CDKN2D, NTRK1, CD74, GDF15, CCL20, GPR55, SPRY4, DAB2IP, LAPTM5, CTCFL, VEGFA, PER2, CENPE, DBNDD2, CRLF1, MAD2L1
16	reproductive structure development	4,2	CDKN1C, CEBPB, SYCP2, SERPINE2, CCNF, LEF1, TNC, HOXD13, ARRB2, BRCA2, HIF1A, CYP19A1, C3, CYP27B1, BRIP1, CCND1 , HES1, PHLDA2, E2F8, PLK4, NTRK1, RRM1, TFAP2C, MME, VDR, TESC, LIF, FANCA, INHBA, FANCG, TBX3, VEGFA, ADAM19, IL1A, SNAI1, PTX3
17	negative regulation of programmed cell death	4,1	DRAXIN, NRP1, ITPRIP, SYCP2, KIF14, TNFAIP3, ARRB2, BRCA1, ICAM1, IL27RA, CCND1 , ADORA1, PDK4, TIMP1, TGM2, HELLS, PRKCH, SPHK1, TENT5B, IL1A, MAG, IL1B, MIR17HG, ARHGEF2, BIRC3, CSF1R, CEBPB, LEF1, AMIGO2, CORO1A, HIF1A, MALT1, AURKB, AURKA, TERT, DCPS, CDKN2D, NTRK1, BARD1, CD74, STIL, EGR3, ATAD5, DDIAS, XRCC2, LAPTM5, HMGA2, TBX3, VEGFA, NR4A1, KRT18, CD28, SNAI1, HDGF, EVI2B, CRLF1, MAD2L1
18	regulation of viral process	4,0	TOP2A, CSF1R, CIITA, CXCL8, ZDHHC20, LEF1, TNFAIP3, PTPN22, KPNA2, MICB, CD74, APOBEC3D, APOBEC3G, HMGA2, ISG20, FAM111A, PLSCR1, RNF125, AIM2, TRIML2, IL1B, CD28, PTX3, APOBEC3A, BIRC3, APOBEC3B
19	regulation of apoptotic process	4,0	TOP2A, DRAXIN, NRP1, ITPRIP, SYCP2, KIF14, TNFAIP3, ARRB2, BRCA1, CTSS, ICAM1, IL27RA, CCND1 , RASSF5, CHEK2, MYB, ADORA1, PDK4, TNFAIP8L1, TIMP3, TNFRSF8, CTLA4, TIMP1, PHLDA2, TGM2, MAP3K5, HELLS, PRKCH, SPHK1, ACTN1, TRAF1, TNFRSF1B, TENT5B, OLFM1, ALDH1A3, IL1A, MAG, MELK, IL1B, MIR17HG, ARHGEF2, BIRC3, CSF1R, CEBPB, LEF1, AMIGO2, CORO1A, HIF1A, MALT1, AURKB, AURKA, TERT, FRZB, CDKN2D, NTRK1, BARD1, CD74, STIL, EGR3, ATAD5, DDIAS, TNFSF15, VDR, XRCC2, DAB2IP, LAPTM5, HMGA2, TNFRSF10B, TNFRSF10A, INHBA, TBX3, VEGFA, NET1, NR4A1, KRT18, CD28, ANP32E, SNAI1, HDGF, EVI2B, CRLF1, MAD2L1, TP73
20	immune system development	3,9	CDKN1C, TOP2A, ANKLE1, NRROS, NCAPG2, TNFAIP3, PTPN22, BRCA2, IL27RA, CYP26B1, UBASH3B, EXO1, MYB, HOXA3, CTLA4, HLA-DOA, IL11, IL4R, TESC, ACTN1, ESCO2, FOS, IL1A, CEACAM1, MELK, FANCD2, IRF1, IL1B, MIR17HG, FAM20C, RAB7B, EPHA2, CSF1R, CEBPB, RBM47, TCF7, LEF1, SLC1A5, HIF1A, MALT1, HLX, NSD2, SLAMF8, HES1, SH2B3, NTRK1, CCR1, CMTM7, CD74, EGR3, ATAD5, GPR55, LIF, FANCA, INHBA, VEGFA, CDK6, CD28, HLA-DRA, EVI2B, EZH2
21	phosphorylation	3,9	DGKG, MYLK2, DGKE, PRAG1, CCNF, BUB1B, MYLK3, ICAM1, CCND1 , UBASH3B, CHEK2, CHEK1, PDK4, ADORA1, EPHB2, MAP3K5, CDK5RAP2, PRKCH, BORA, VRK1, PASK, HASPIN, CDC25C, CDC25A, MELK, CCNE2, CMPK2, TRIB3, EPHA2, CSF1R, BLM, RASAL1, AK5, PKMYT1, HIF1A, C3, CCNB1, SLAMF8, CLSPN, FAM83D, CCR1, PLK4, TNFSF15, LIF, TNFRSF10B, HMGA2, TNFRSF10A, INHBA, MAP3K15, CDK6, HOOK1, CCDC88C, CSPG4, CHRFAM7A, EZH2, TP73, CDKN1C, NRP1, CIITA, KIF14, TNFAIP3, PTPN22, ARRB1, ARRB2, C1QTNF1, PBK, PMEPA1, TIMP3, IL11, SPHK1, CTDSPL, EMP2, TRAF1, MASTL, CIT, CCNA1, IL1A, CEACAM1, PSRC1, ADORA2B, IL1B, ITPKA, FAM20C, ARHGEF2, AVPI1, OPRD1, DTYMK, PKN3, CAMKK1, MALT1, AURKB, AURKA, HES1, SH2B3, BUB1, MAPK4, CDKN2D, NTRK1, CD74, GDF15, CCL20, GPR55, SPRY4, DAB2IP, LAPTM5, MAPK12, MAPK13, VEGFA, CENPE, DBNDD2, CRLF1, PTPN3
22	regulation of cell-cell adhesion	3,9	CEBPB, SERPINE2, MEGF10, TCF7, LEF1, PTPN22, CORO1A, MALT1, IL27RA, CYP26B1, C1QTNF1, UBASH3B, FCHO1, HLX, PODXL, MYB, RAC2, CTLA4, HES1, HLA-DOA, SH2B3, CD74, EGR3, IL4R, LAPTM5, FANCA, TNFRSF1B, VEGFA, ADAM19, IL1A, CEACAM1, NR4A3, FANCD2, IRF1, IL1B, CD28, HLA-DRA, HLA-DQB1
23	positive regulation of macromolecule metabolic process	3,6	MYLK2, EHF, DSCC1, TNC, ICAM1, ELK3, CCND1 , RASSF5, CHEK2, DPF1, CHEK1, MYB, ADORA1, EPHB2, MAP3K5, HOXA4, CDK5RAP2, BORA, ZBTB38, ACTN1, PASK, ADAM19, OLFM1, TRIB3, DNA2, MAML3, TRABD2A, CSF1R, BLM, CDCA2, CDCA4, HIF1A, RAD51AP1, C3, TERT, WBP2NL, HIVEP3, CLSPN, BARD1, FANCI, CCR1, TFAP2C, AFAP1L2, EGR3, FUS, TNFSF15, VDR, ALYREF, LIF, TNFRSF10B, HMGA2, TNFRSF10A, INHBA, NR1D1, TRERF1, NR4A1, NR4A3, CDK6, UBE2S, MAFF, CD28, SNAI1, CSPG4, CHRFAM7A, PHF19, EZH2, TP73, CDKN1C, TOP2A, MESP1, NRP1, CIITA, CXCL8, SUV39H1, KIF14, TNFAIP3, PTPN22, ARRB1, ARRB2, BRCA1, BRCA2, EFCAB7, CTSS, CYP26B1, C1QTNF1, RFC5, IL11, FOXD2, RFC3, RBM14, RFC4, SPHK1, TESC, CKAP2, ETV1, EMP2, TRAF1, FOS, TENT5B, MASTL, TNFRSF1B, ETV4, IL1A, PLSCR1, DBF4, AIM2, PSRC1, IRF1, IL1B, ARHGEF2, DTL, BIRC3, AVPI1, OPRD1, CEBPB, UHRF1, LEF1, HOXD13, RCHY1, CAMKK1, MALT1, AURKB, AURKA, PSMC3IP, EXOSC9, NSD2, USP1, E2F2, HES1, E2F8, NTRK1, GINS1, CD74, ATAD5, GDF15, CCL20, GINS3, F12, GPR55, ATAD2, DAB2IP, LAPTM5, TBX3, MAPK12, CTCFL, VEGFA, PER2, CENPE, KLF5, CENPJ, NFIC, HDGF, CRLF1, ZNF850
24	DNA damage checkpoint	3,4	BLM, TIPIN, BRIP1, CCND1 , FANCD2, CHEK2, CHEK1, HMGA2, BRCA1, CLSPN, DTL, TICRR
25	movement of cell or subcellular component	3,4	MYLK2, PRAG1, SERPINE2, WIPF1, TNC, ICAM1, IL27RA, ADORA1, C3AR1, KIF21B, FBXO5 , EPHB2, PHLDA2, SCN1B, SEMA6B, S100A2, IGFBP5, ACTN1, DEPDC1B, DAAM2, LGALS12, KIF20B, EPHA2, CSF1R, SDC3, WDR62, HIF1A, CYP19A1, CORO1A, TERT, PODXL, SLAMF8, LPXN, FAM83D, CCR1, EGR3, ARHGEF39, RAB27B, NET1, FERMT1, NR4A1, CXCL11, NR4A3, CDK6, CCDC88C, SNAI1, FAT2, RADIL, CSPG4, CD200, MESP1, NRP1, DRAXIN, ROBO4, CXCL8, MEGF10, KIF14, TNFAIP3, KIF12, LAMC2, PTPN22, CXCL1, ARRB2, KIF11, KIF15, ADGRE2, PDE4B, RAC2, SPDL1, TIMP1, SPHK1, ARHGEF16, SHROOM2, KIF24, ETV1, EMP2, KIF22, IL1A, CEACAM1, ACTBL2, IL1B, KIF2C, MNS1, LCP1, ARHGEF2, SSX2IP, DNAH3, KANK1, PKN3, LEF1, SEMA3G, ADCY3, DNAH9, SH3BP1, HES1, NTRK1, CD74, LAMB3, CCL20, DAB2IP, VAX1, VEGFA, CENPE, FMNL1, SAA1, SAA2
26	regulation of mitotic spindle checkpoint	3,2	CCNB1, XRCC3, KNL1, AURKB, MAD2L1, CDK5RAP2
27	chromatin remodeling at centromere	3,2	HELLS, MIS18A, CENPW, OIP5, CENPN, CENPA, ITGB3BP
28	meiosis II	3,1	SGO1, SGO2, STAG3, BUB1B, MASTL, BUB1
29	granulocyte migration	3,1	CD74, CSF1R, CXCL8, CCL20, CXCL1, EMP2, VEGFA, IL1A, ADGRE2, CXCL11, IL1B, SLAMF8, C3AR1, SAA1, PDE4B, RAC2, SAA2
30	mitotic G2/M transition checkpoint	3,0	BARD1, BLM, ORC1, CHEK1, HMGA2, BRCA1, CLSPN, DTL, TICRR

Supp. Table 4

serial number	Rank in PSCAN analysis	Transcription factor	Transcription factor full name	Overrepresentation Score (1/p-value)
**1**	1	TFDP1	Transcription Factor Dp-1	5,8497E+23
**2**	2	E2F6	E2F Transcription Factor 6	8,12486E+17
**3**	18	E2F2	E2F Transcription Factor 2	1,30583E+11
**4**	29	E2F8	E2F Transcription Factor 8	11339269819
**5**	41	E2F4	E2F Transcription Factor 4	1660732317
**6**	43	E2F1	E2F Transcription Factor 1	1037624256
**7**	81	E2F7	E2F Transcription Factor 7	20181,87909
**8**	99	E2F3	E2F Transcription Factor 3	2643,579407

Supp. Table 5

serial number	Antibody against	Species	Dilution factor	Catalgoue number	Company name
1	FOXM1	Rabbit	1:1000	#20459	Cell Signalling, Frankfurt, Germany
2	Cyclin D1	Rabbit	1:1000	#55506	Cell Signalling, Frankfurt, Germany
3	phoshpo-RB S780)	Rabbit	1:1000	#9307	Cell Signalling, Frankfurt, Germany
4	p21	Rabbit	1:1000	#2947	Cell Signalling, Frankfurt, Germany
5	Cyclin E2	Rabbit	1:1000	#4132	Cell Signalling, Frankfurt, Germany
6	cMYc	Rabbit	1:1000	#5605	Cell Signalling, Frankfurt, Germany
7	DUSP4	Rabbit	1:1000	#5149	Cell Signalling, Frankfurt, Germany
8	ERK5	Rabbit	1:8000	#E1523	Sigma-Aldrich, Darmstadt, Germany
9	HA	Rabbit	1:1000	#H6908	Sigma-Aldrich, Darmstadt, Germany
10	a-tubulin	Mouse	1:10000	#T5168	Sigma-Aldrich, Darmstadt, Germany
11	ZEB1	Rabbit	1:1000	#HPA027524	Sigma-Aldrich, Darmstadt, Germany
12	ZEB2	Rabbit	1:1000	#HPA003456	Sigma-Aldrich, Darmstadt, Germany
13	p27	Mouse	1:1000	#610241	BD Biosciences, Heidelberg, Germany
14	Ecadherin	Mouse	1:1000	#610181	BD Biosciences, Heidelberg, Germany
15	Ncadherin	Mouse	1:1000	#sc-271386	Santa Cruz Dallas, Texas, USA
16	EMI1	Mouse	1:1000	#37-6600	ThermoFisher Scientific Darmstadt, Germany
17	MITF	Mouse	1:1000	#ab12039	Abcam, Cambridge, UK
18	SOX10	Rabbit	1:1000	#383R-18	Medac, Wedel, Germany
19	MEK5	Rabbit	1:1000	#AB3184	Merck Millipore, Darmstadt, Germany

Supp. Table 6

serial number	Gene	Forward primer (5'-3')	Reverse primer (5'-3')
1	*FOXM1*	CGTCGGCCACTGATTCTCAAA	GGCAGGGGATCTCTTAGGTTC
2	*CCND1*	TCTACACCGACAACTCCATCCG	TCTGGCATTTTGGAGAGGAAGTG
3	*GAPDH*	CCACCCATGGCAAATTCC	GATGGGATTTCCATTGATGACA
4	*CCNE2*	TCCTTCACCTTTGCCTGATT	CCTCATCTGTGGTTCCAAGTC
