# Supplementary material for: MEK5/ERK5 inhibition sensitizes NRAS-mutant melanoma to MAPK-targeted therapy by preventing Cyclin D/CDK4-mediated G1/S progression
Source: Cell Death Dis. 2025 Oct 6;16(1):689. doi: 10.1038/s41419-025-08036-7 (PMC12501213; doi:10.1038/s41419-025-08036-7)

Figure 1D FM79

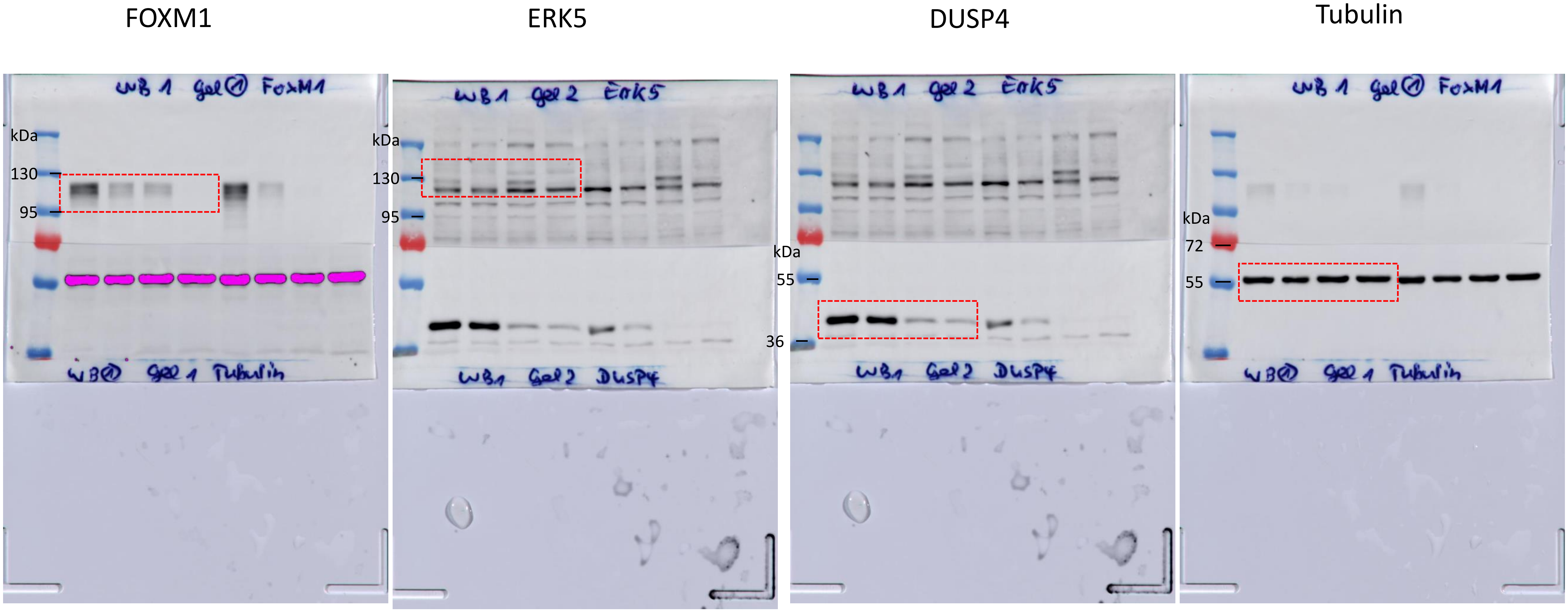

Figure 1D M26

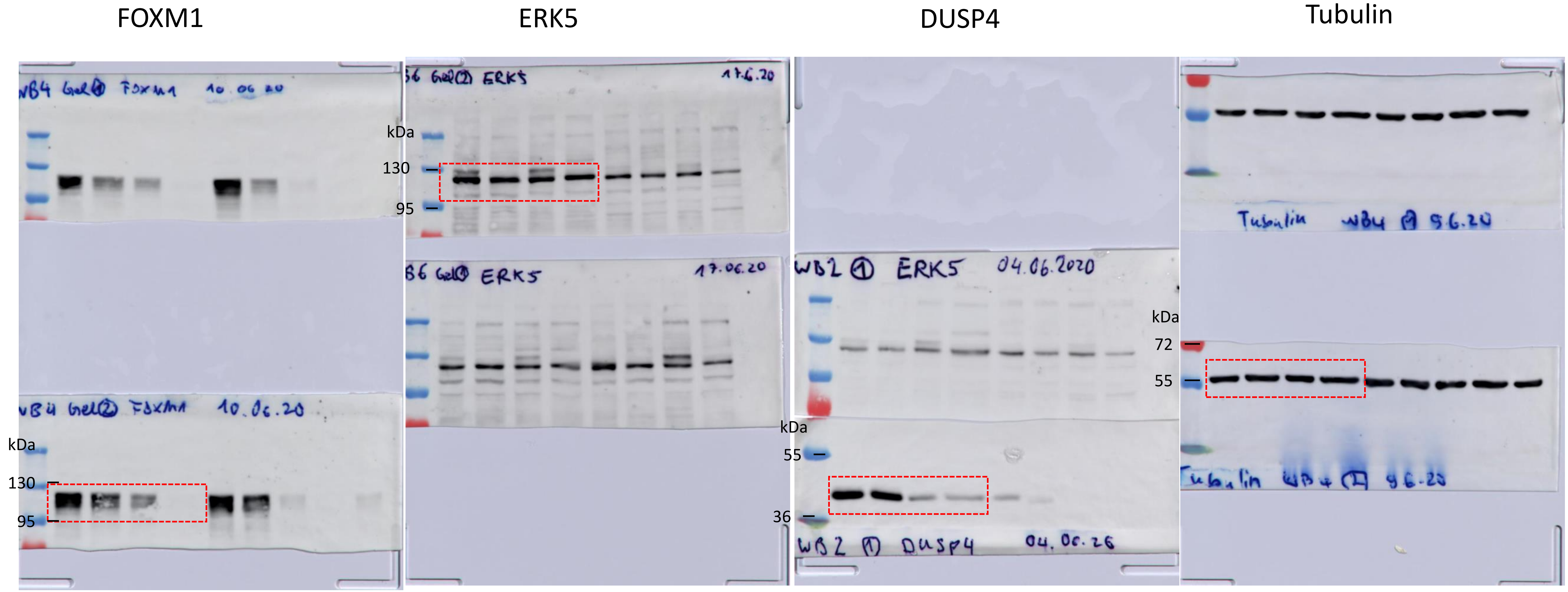

Figure 1D BLM

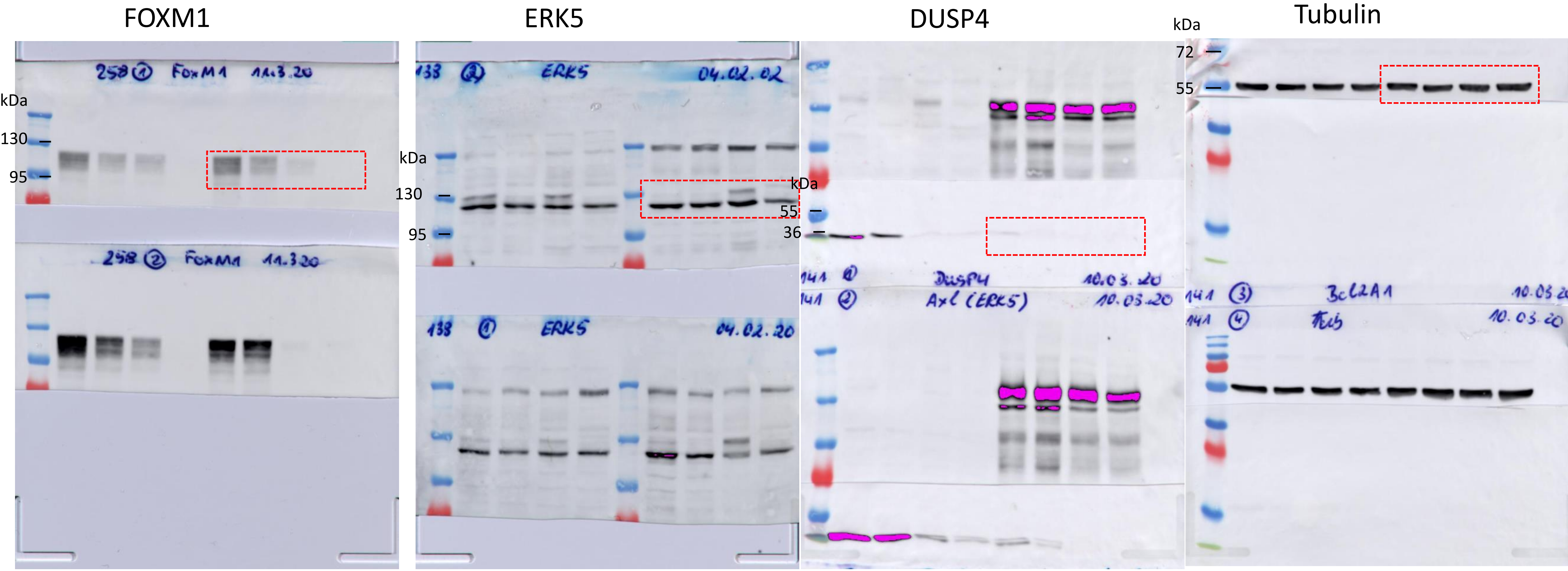

Figure 1D MaMel26a

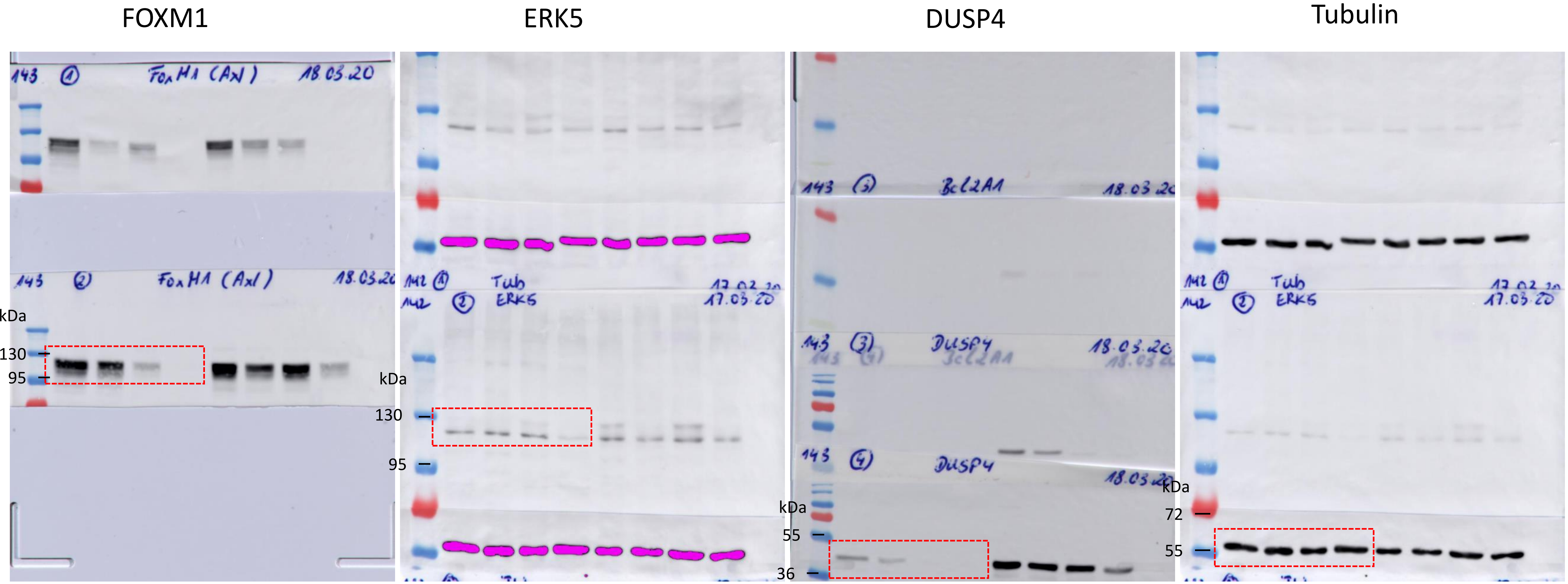

Figure 2C M26

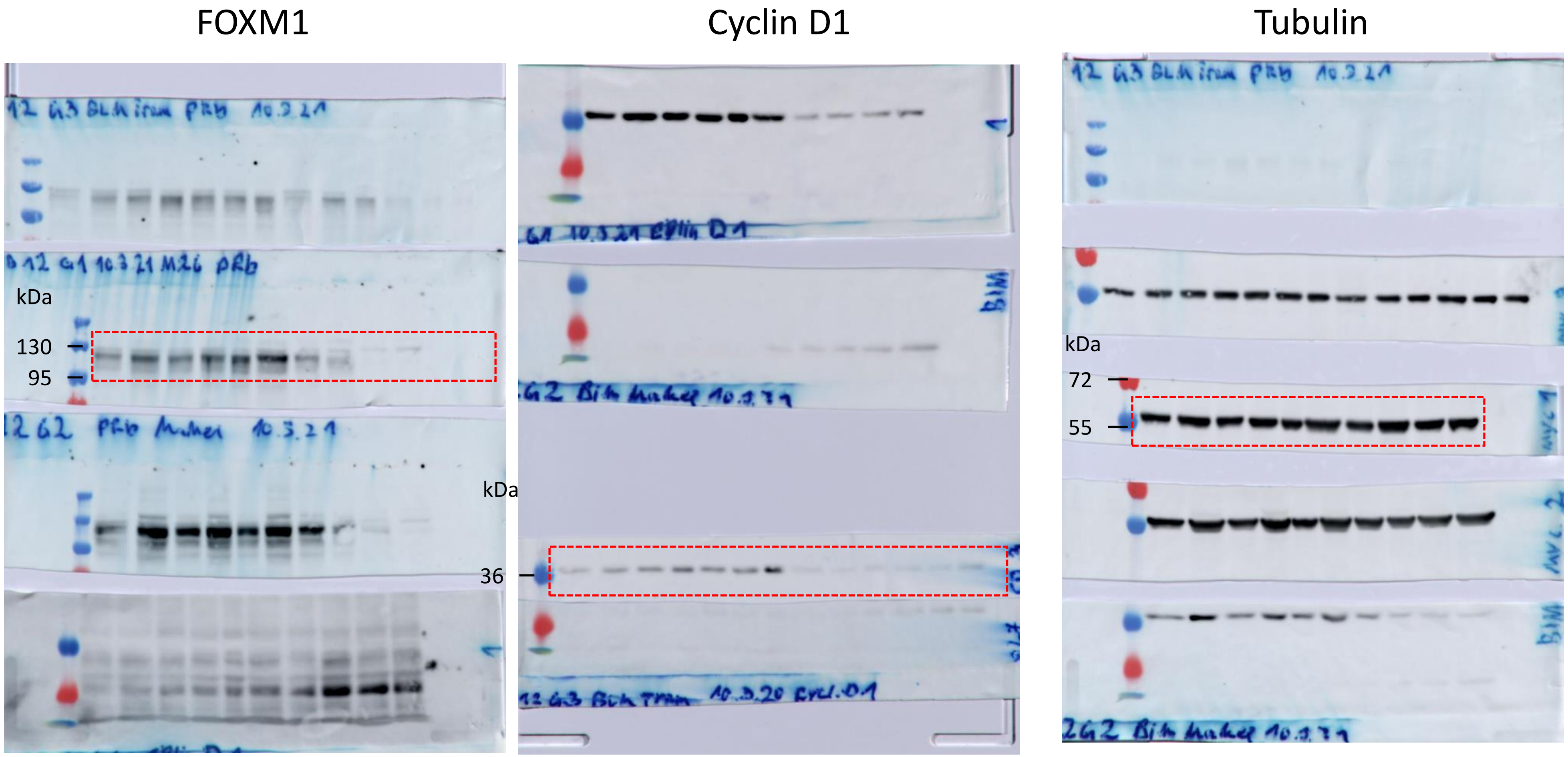

Figure 2D BLM

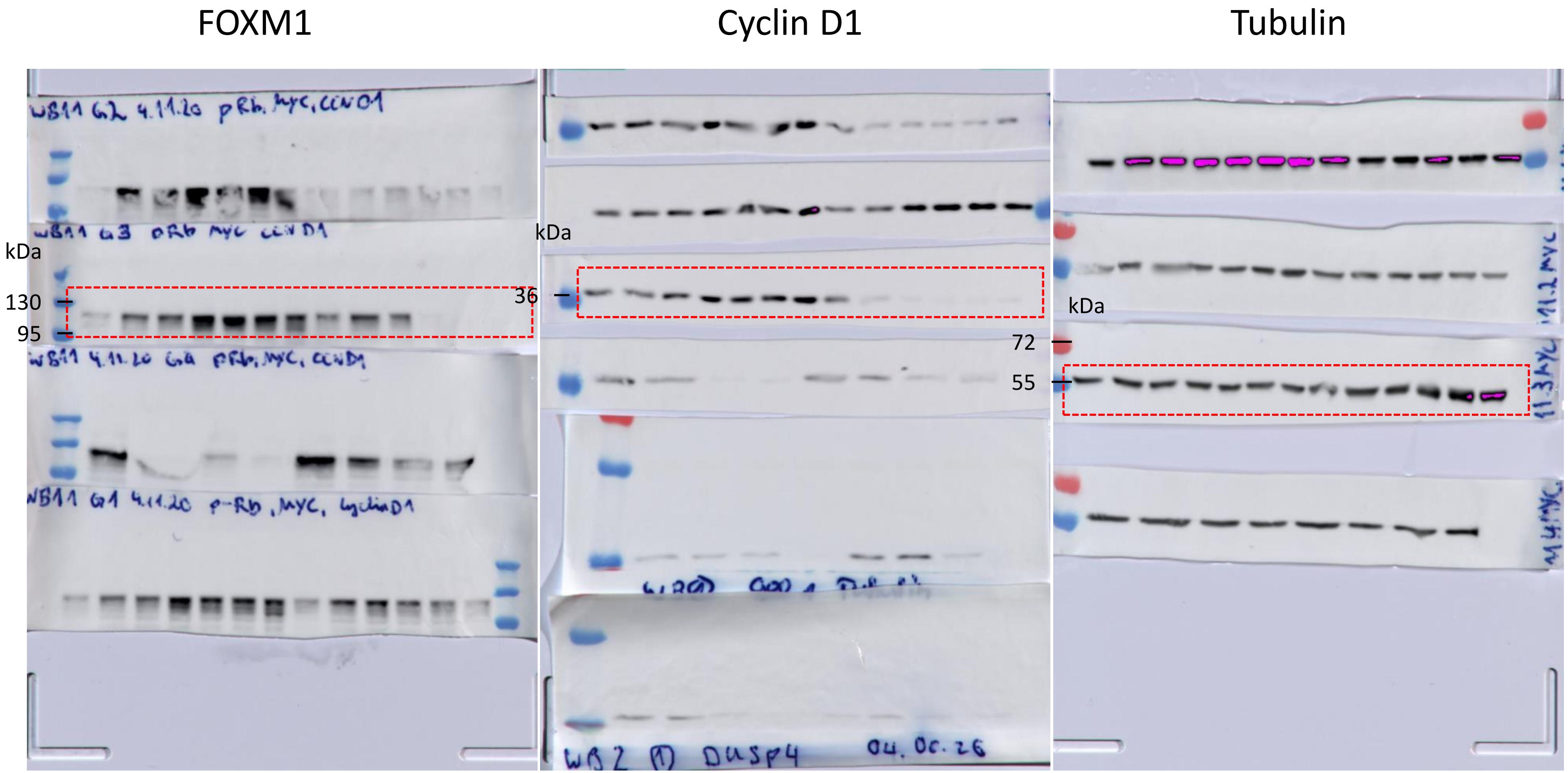

Figure 3B

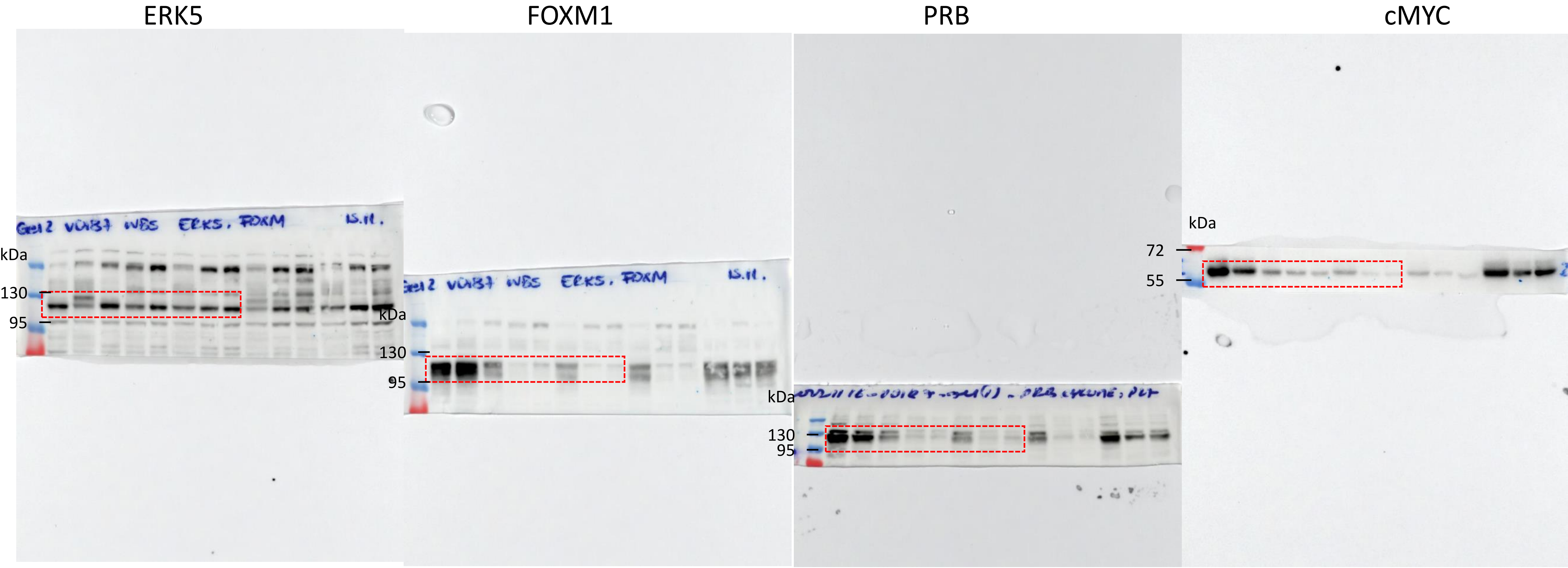

Stripped from FOXM1

Figure 3B Contd.

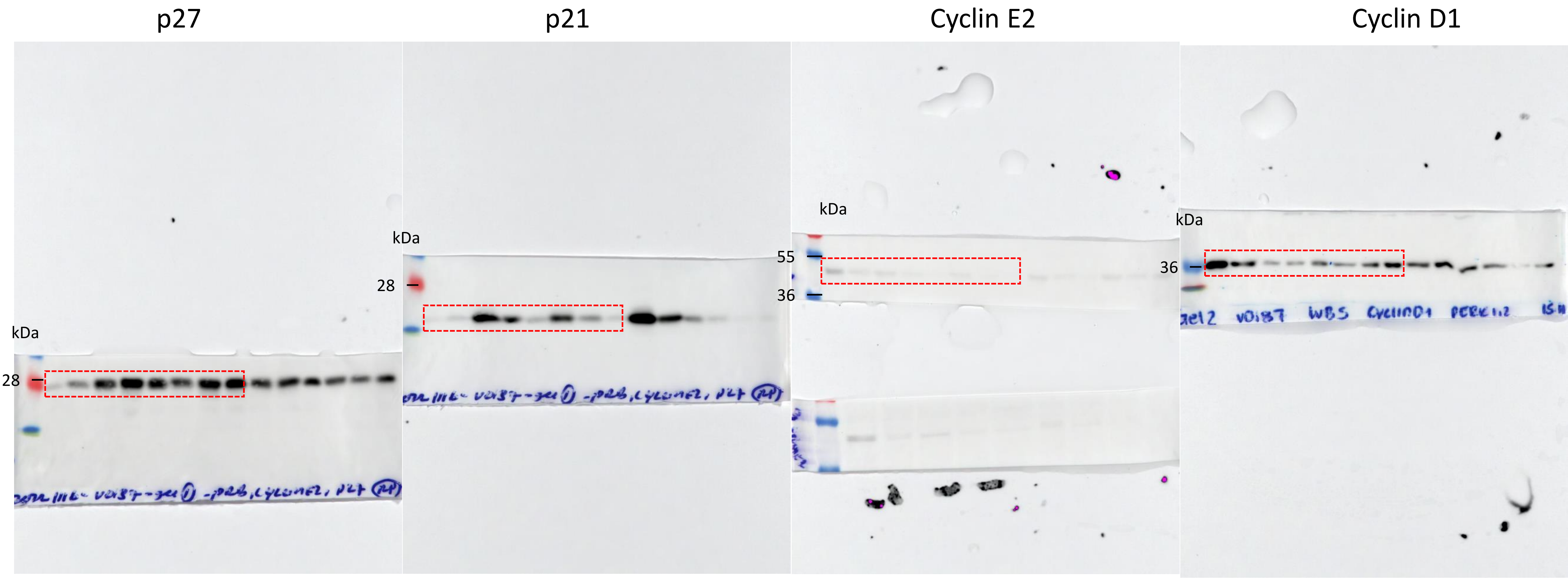

Figure 3B Contd.

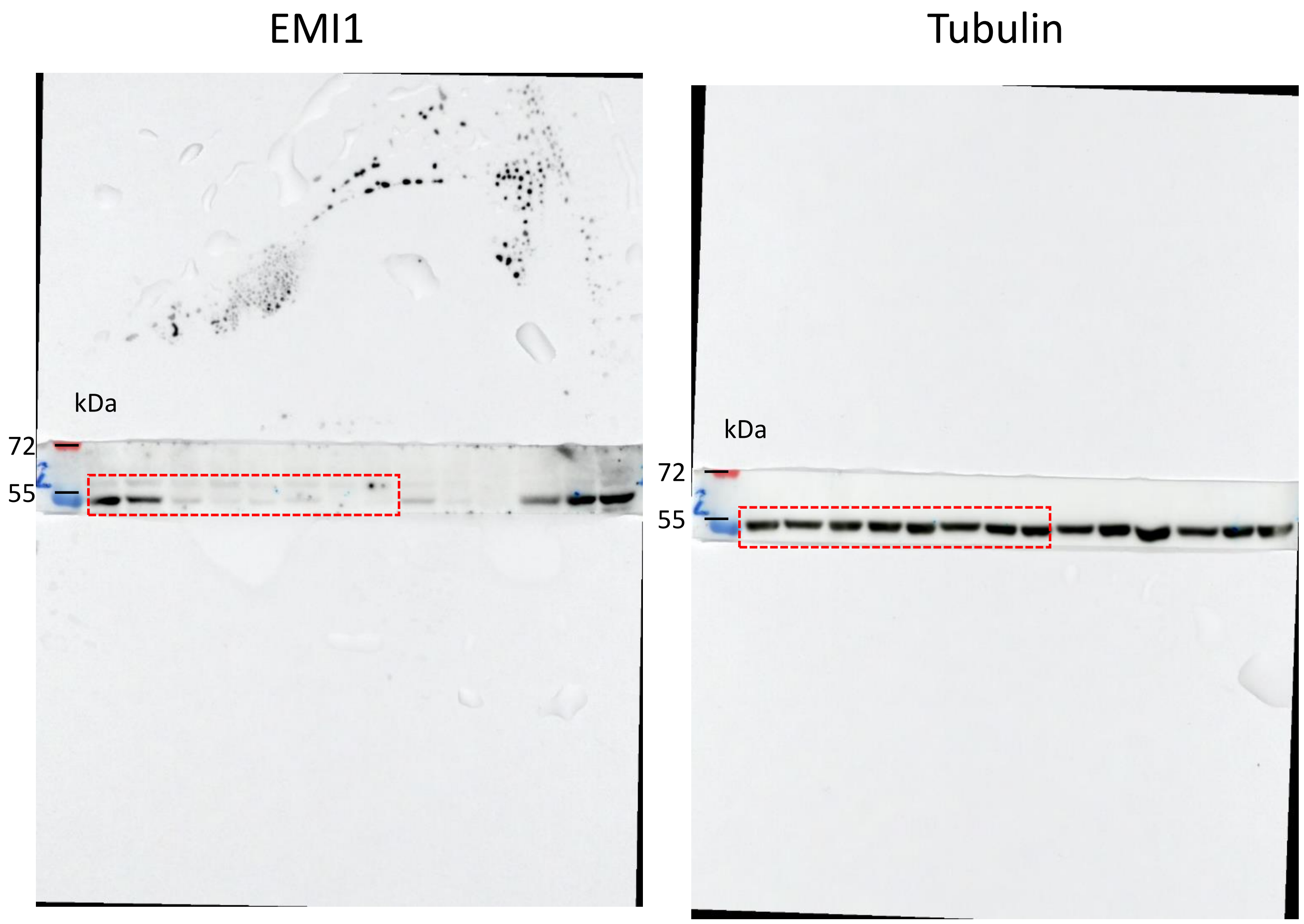

Figure 4A

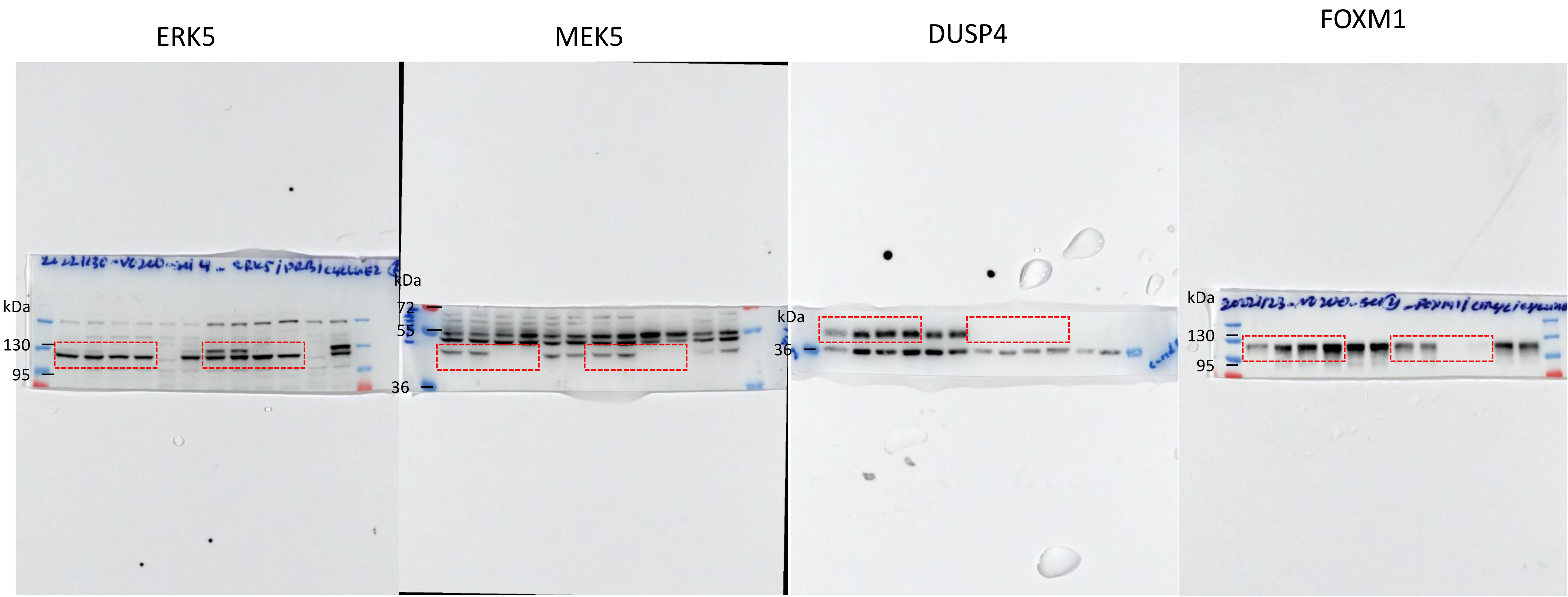

Stripped from CCND1

Figure 4A Contd

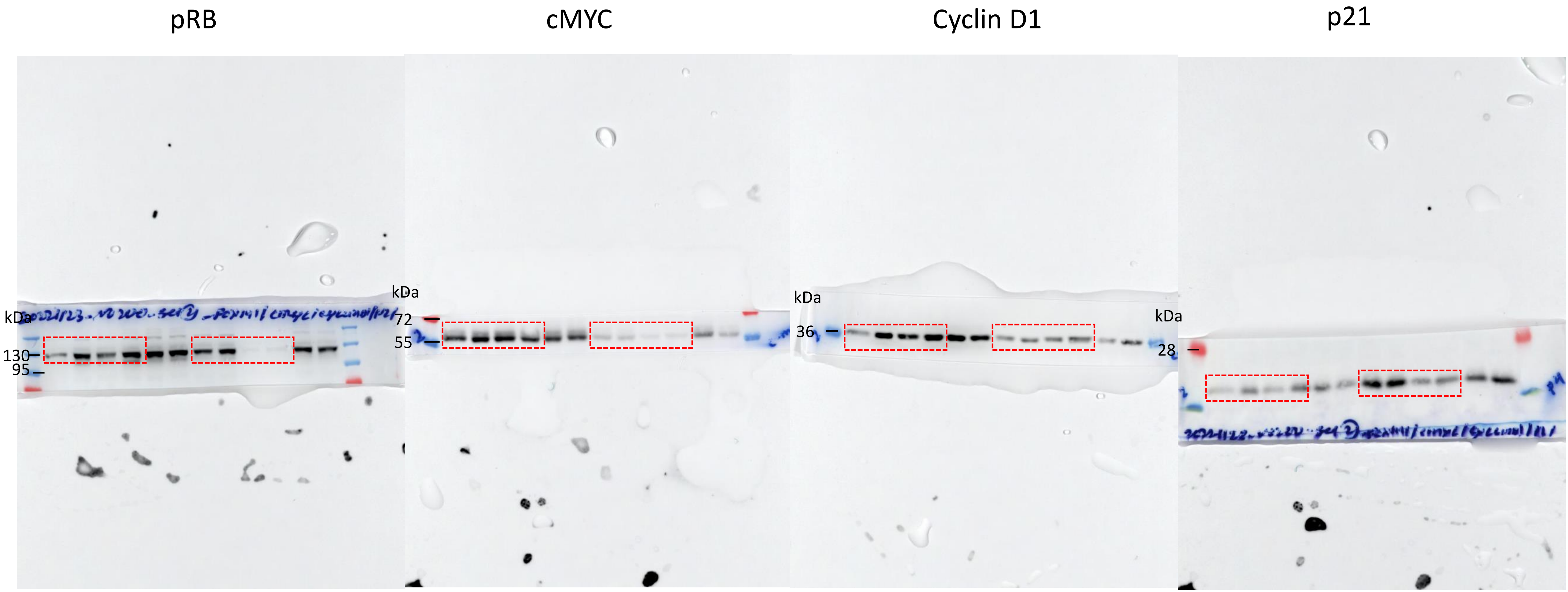

Stripped from FOXM1

Figure 4A Contd

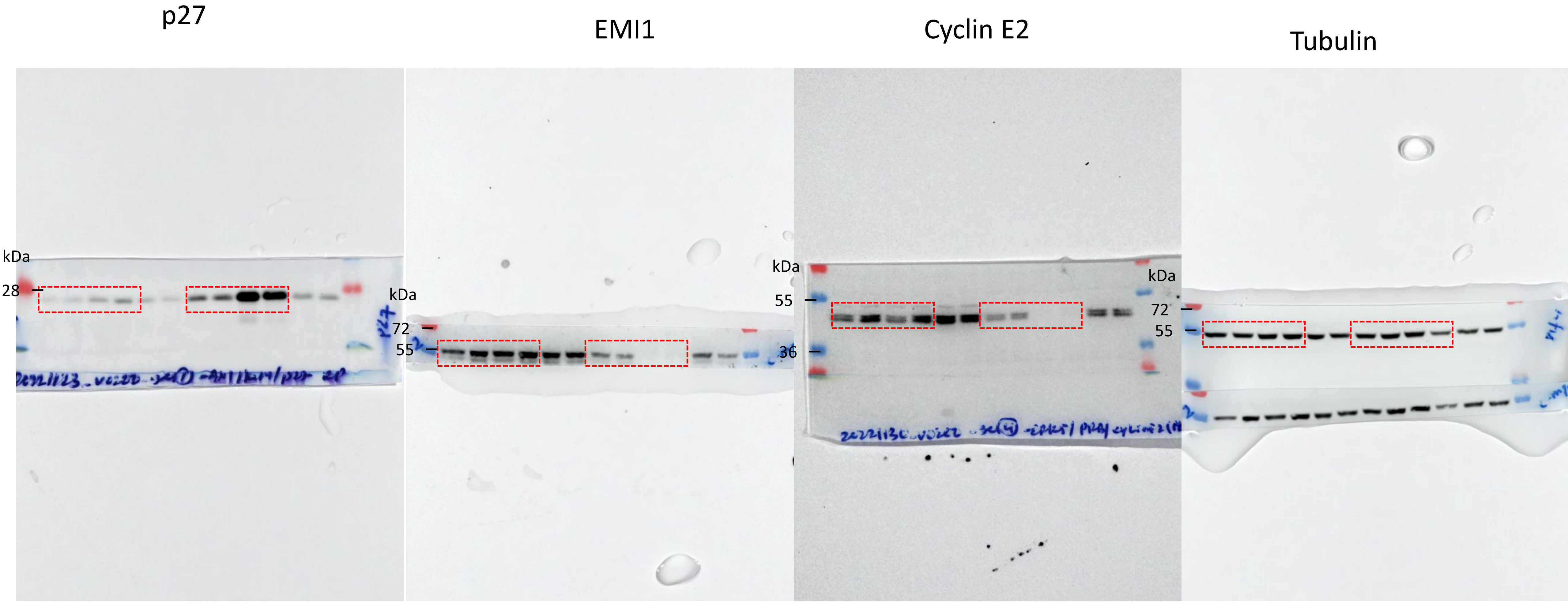

Stripped from cMYC

Figure 5B

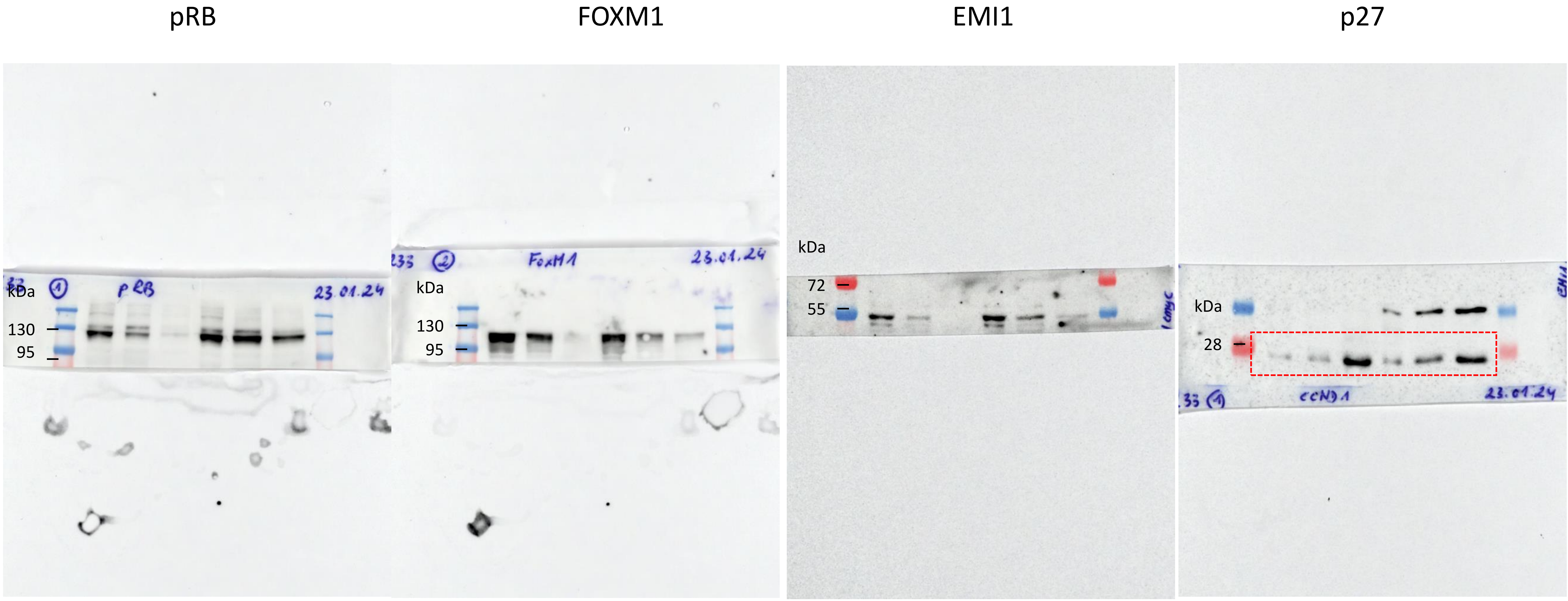

Stripped from cMYC

Stripped from Cyclin D1

Figure 5B contd.

p21

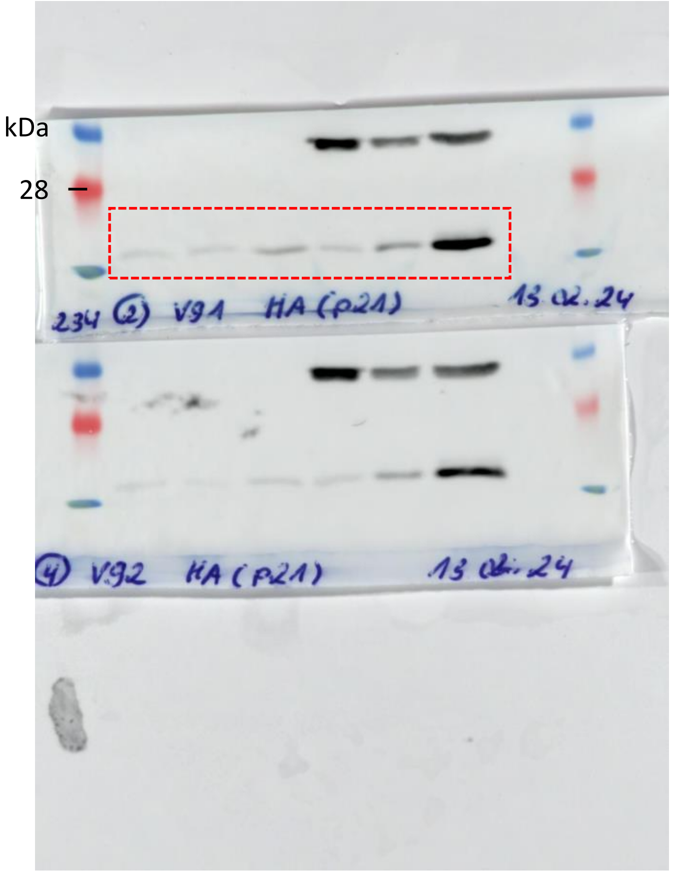

Stripped from HA

Figure 5B contd.

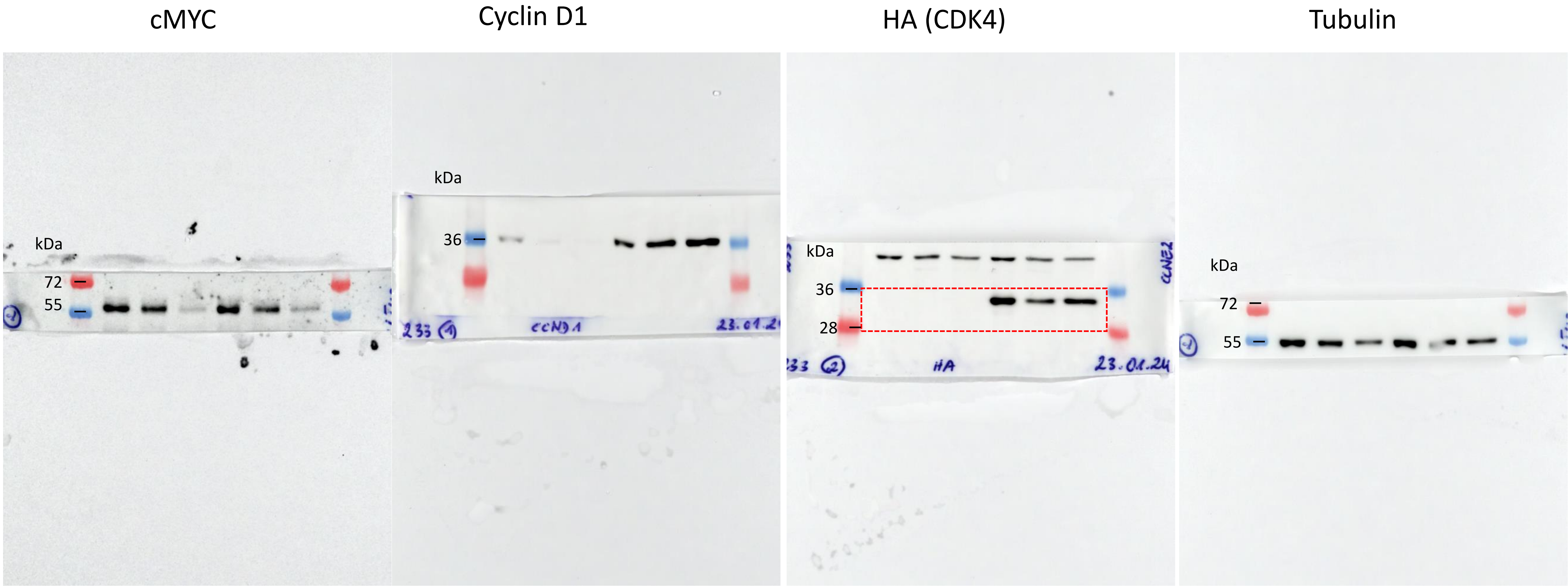

Figure 5D

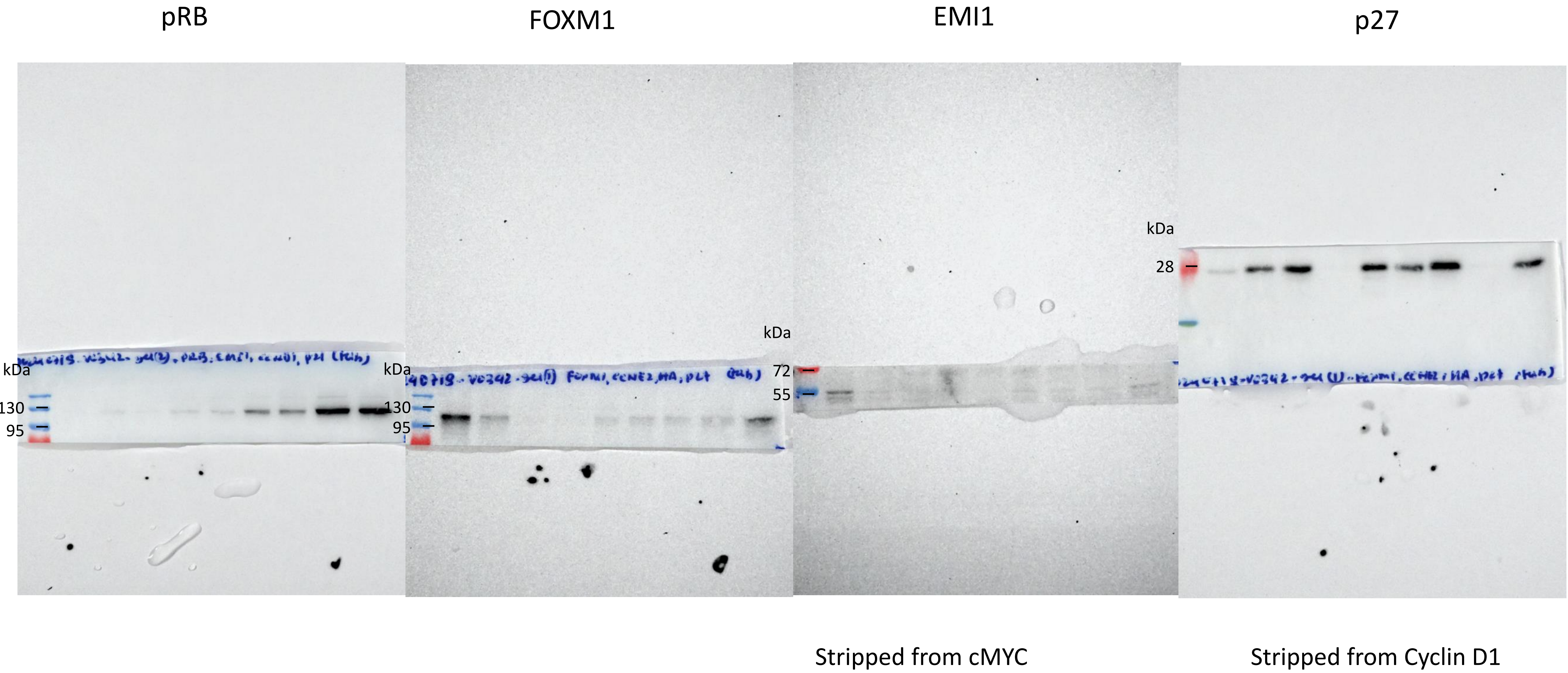
pRB
FOXM1
EMI1
p27
kDa
130
95
kDa
130
95
kDa
72
55
kDa
28
Stripped from cMYC
Stripped from Cyclin D1

Figure 5D contd.

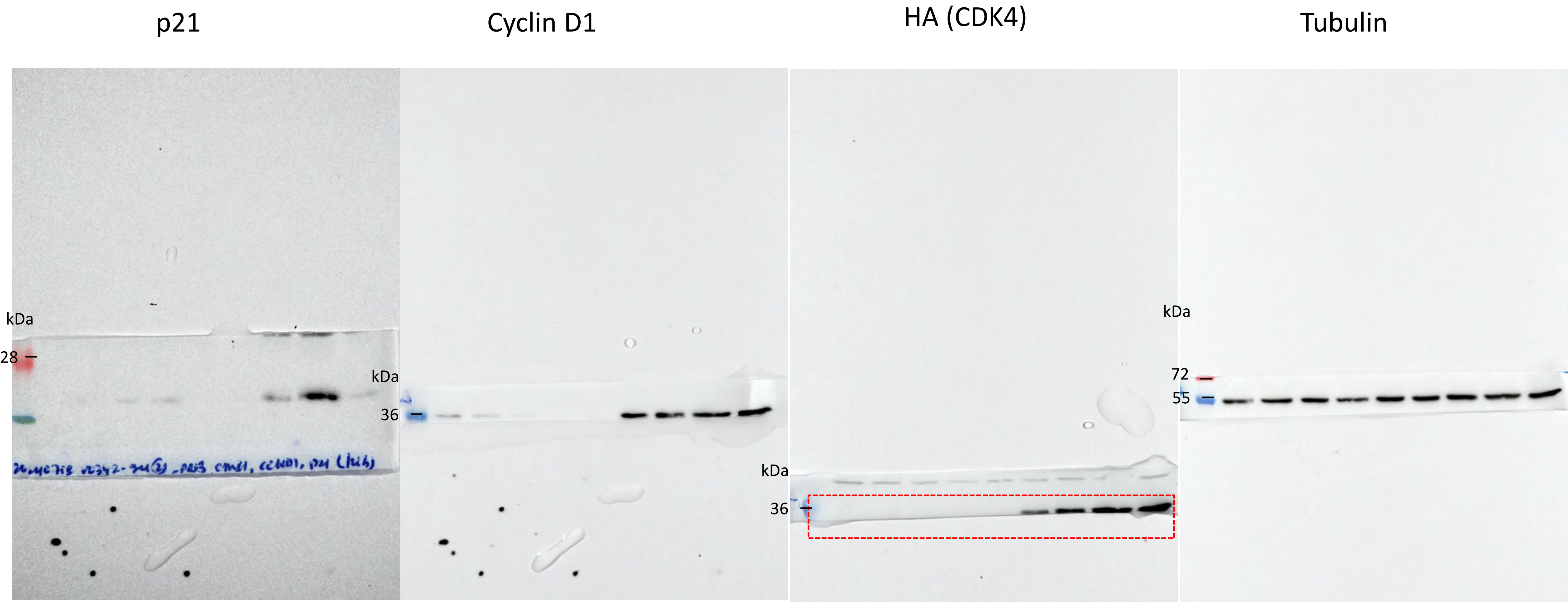

Figure 6B

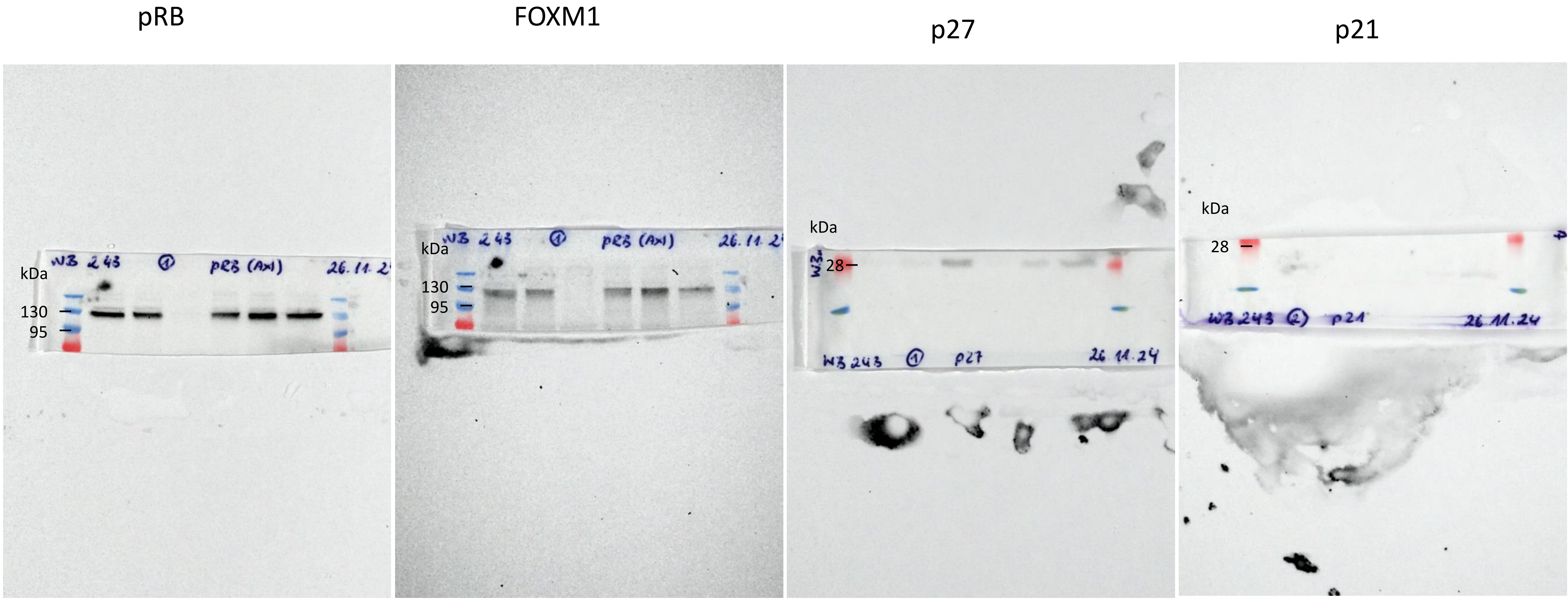

Stripped from pRB

Figure 6B contd.

Cyclin D1

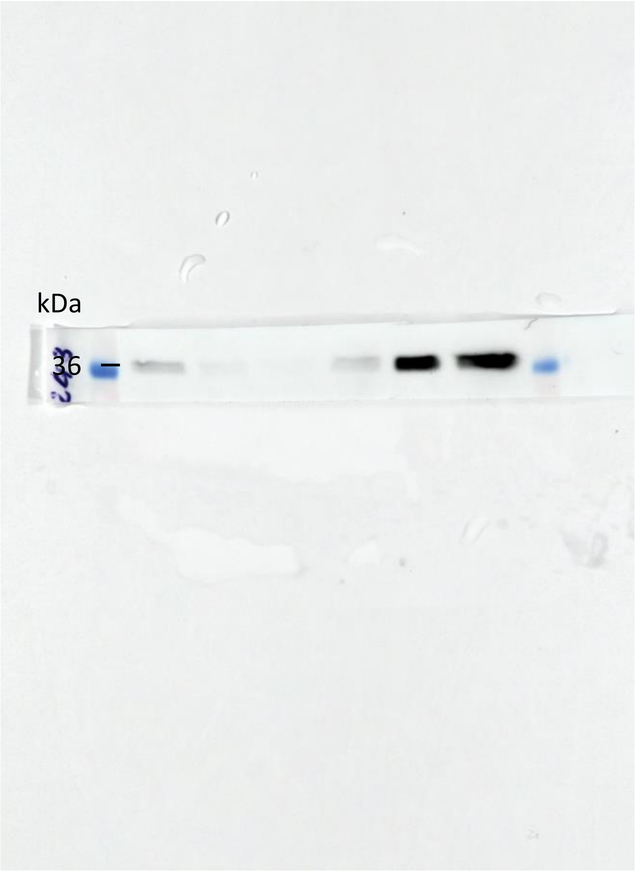

HA (CDK4)

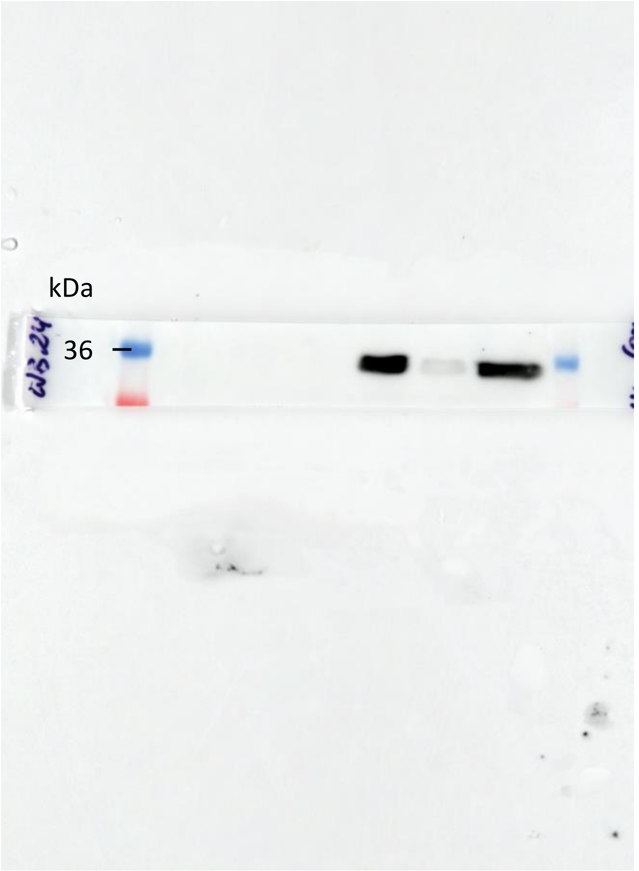

Tubulin

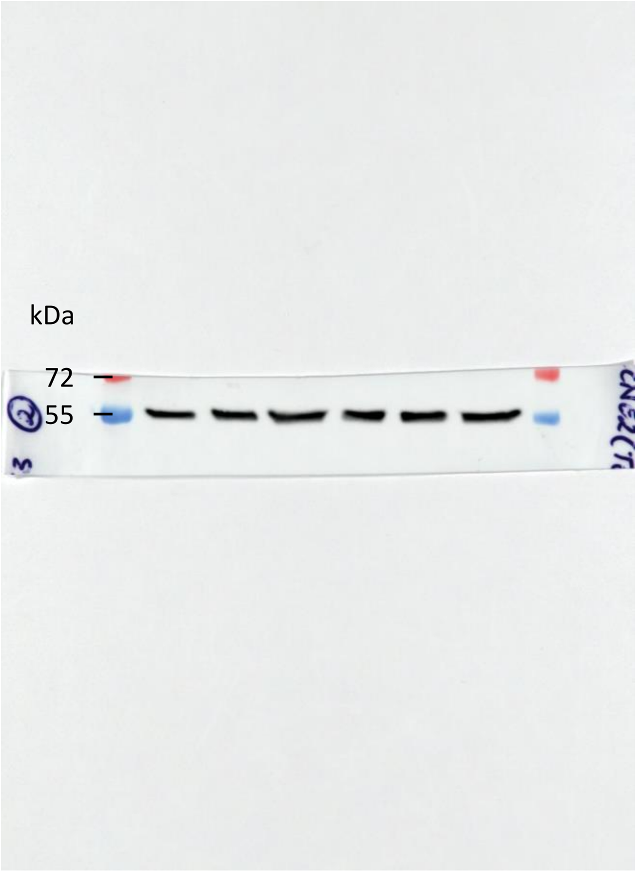

Figure 6D

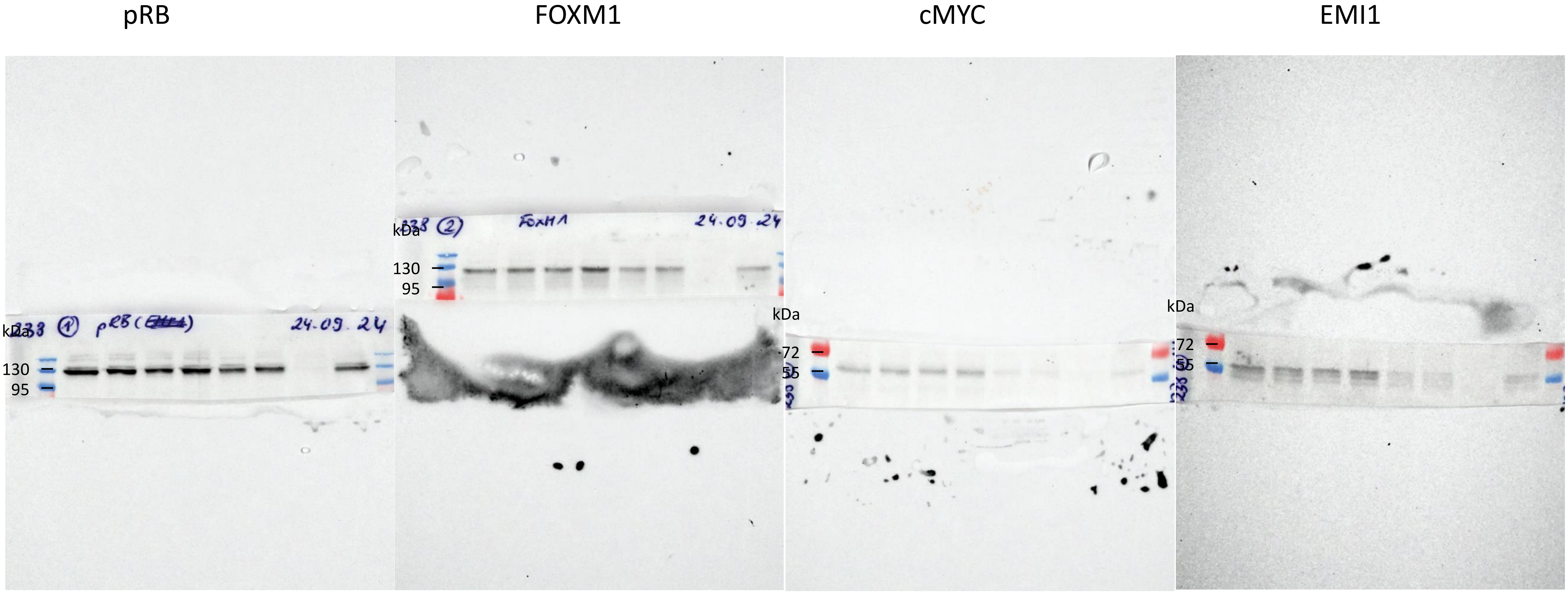
pRB
FOXM1
cMYC
EMI1
kDa
130
95
kDa
130
95
kDa
72
55
kDa
72
55
24.09.24
24.09.24

Figure 6B contd.

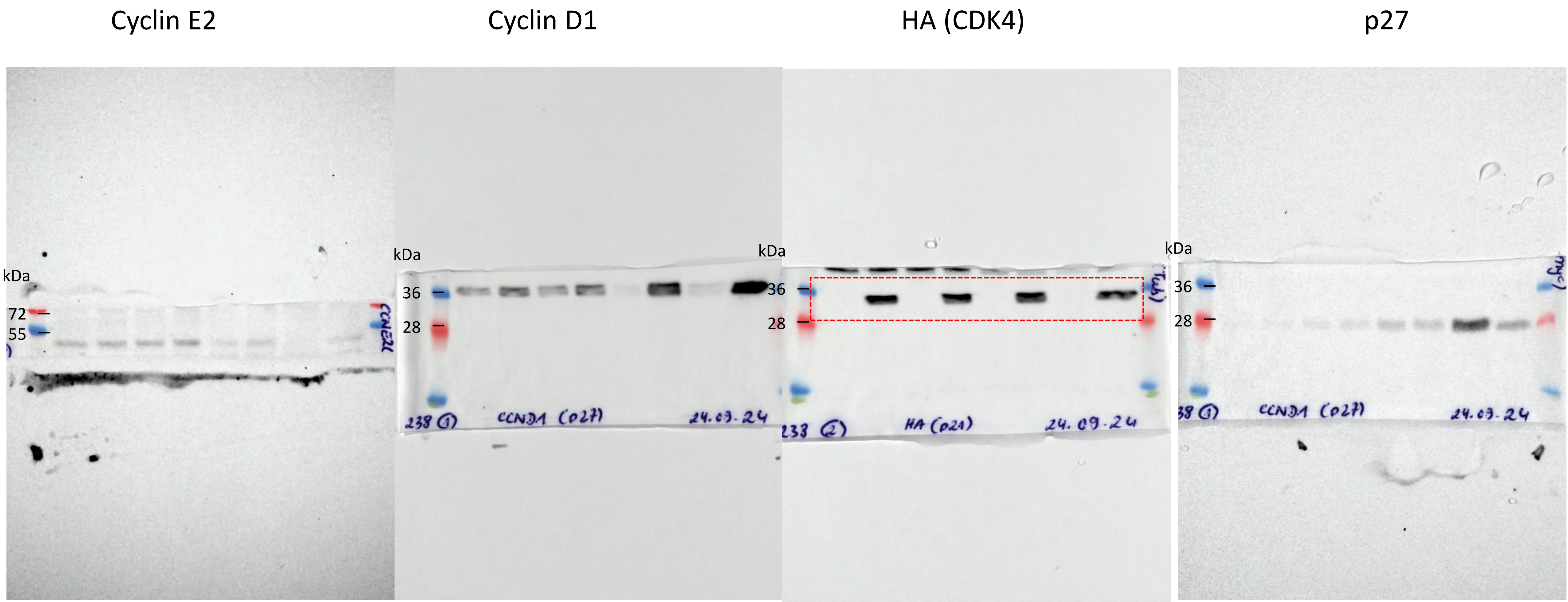

Figure 6B contd.

p21

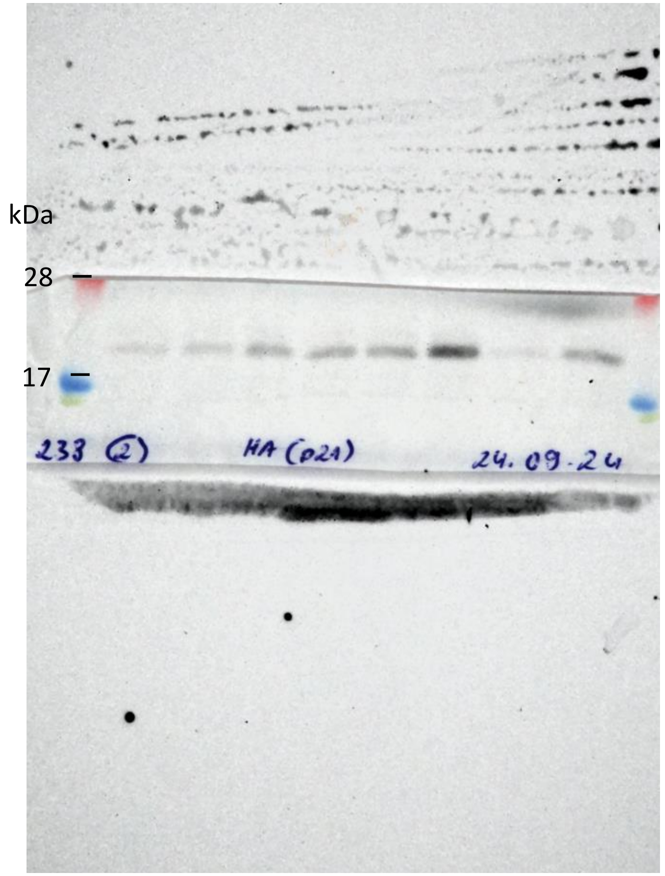

Tubulin

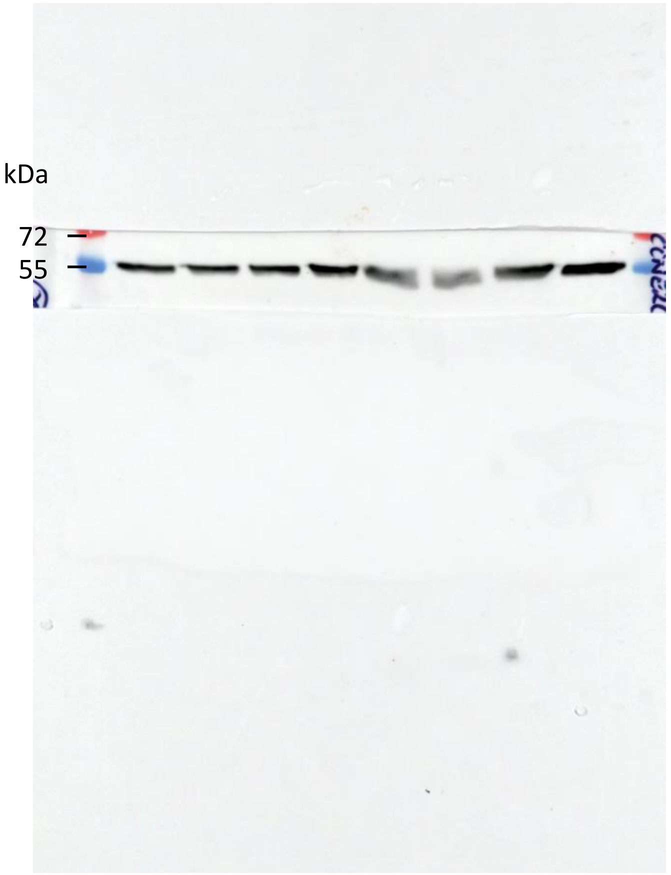

Supp Figure 2D

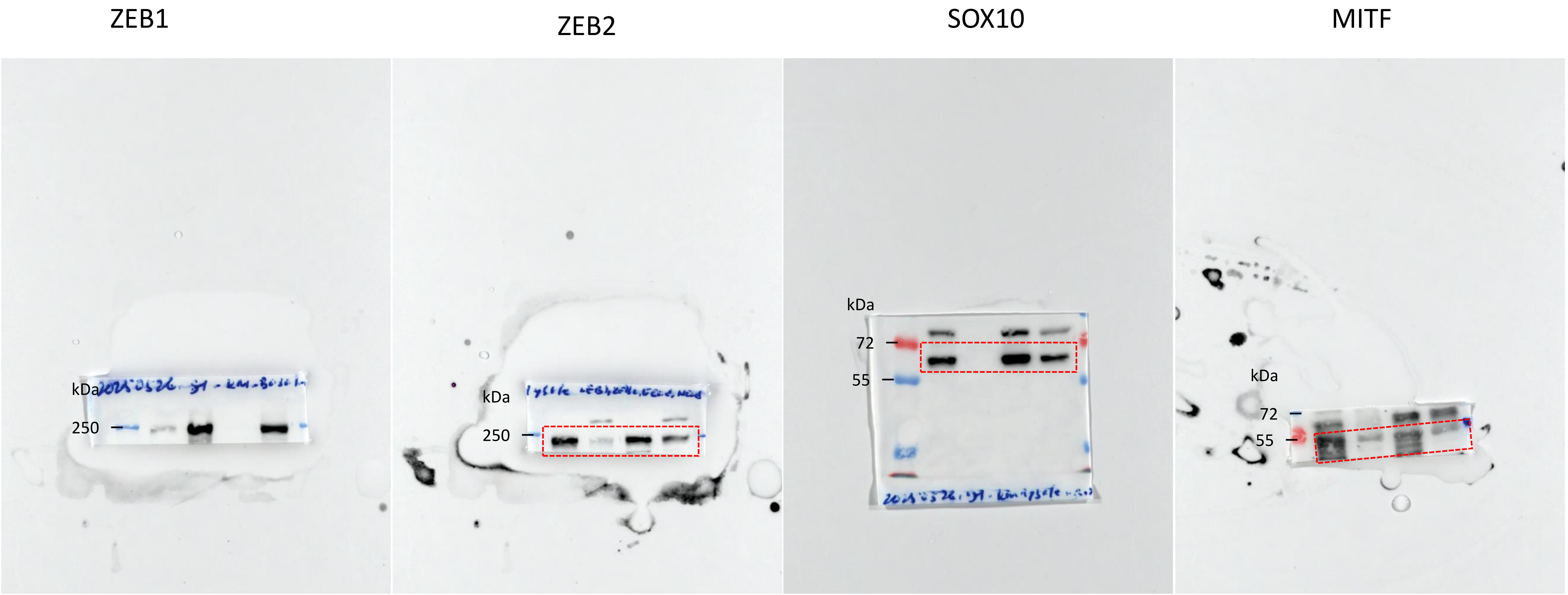

Supp Figure 2D Contd.

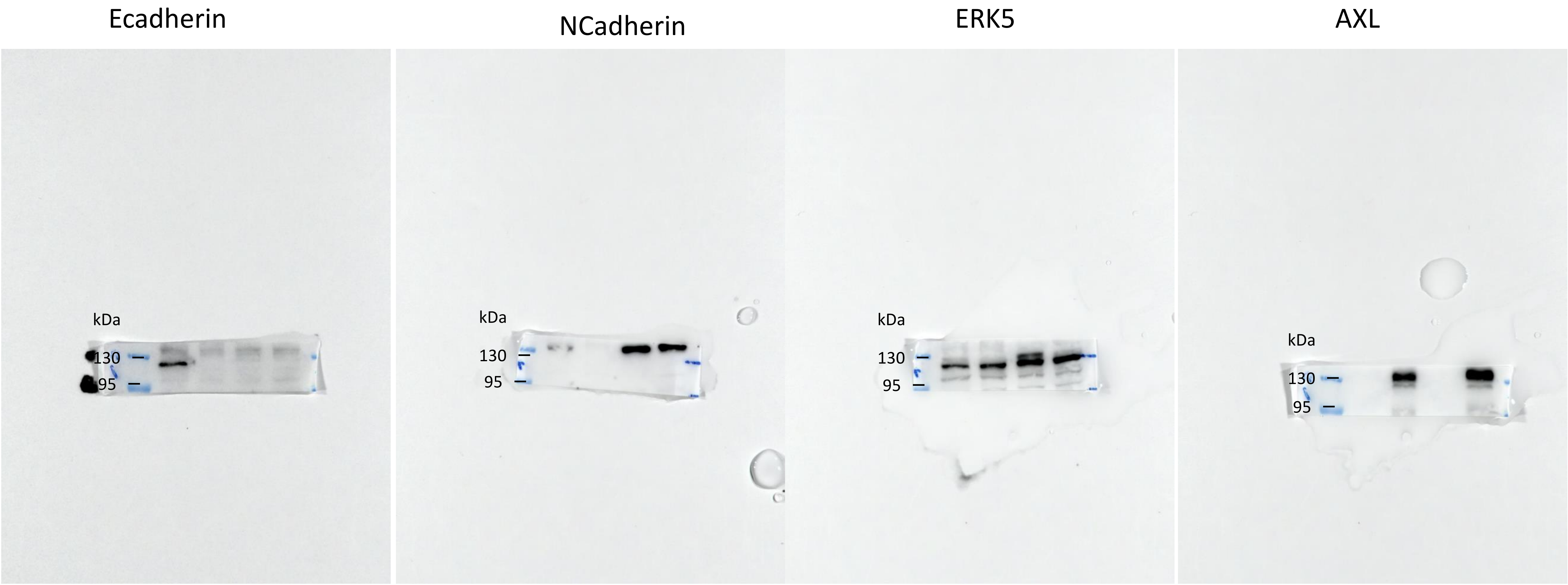

Supp Figure 2D Contd.

Tubulin

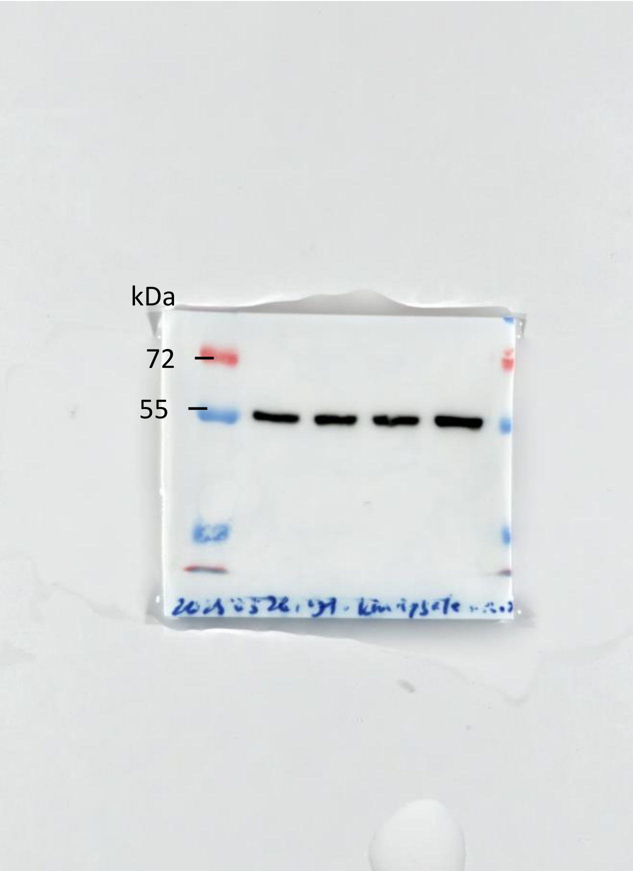

Supp Figure 4B

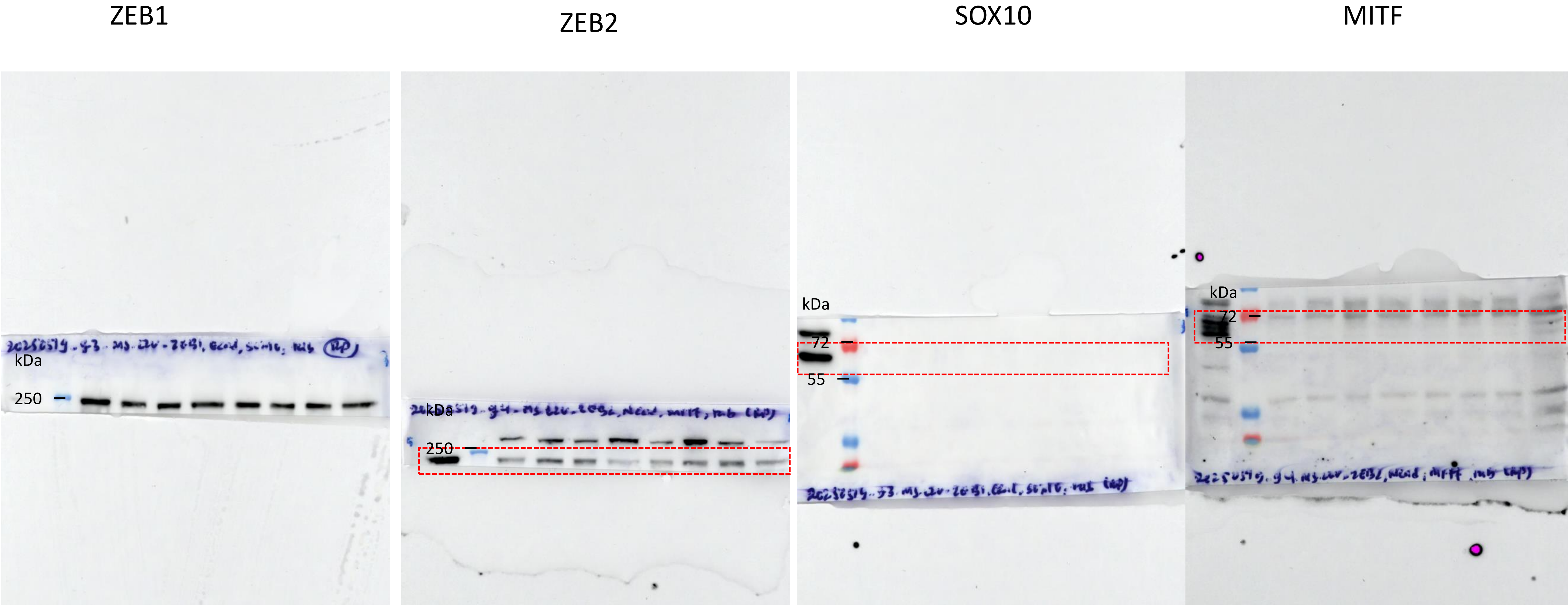

Supp Figure 4B Contd.

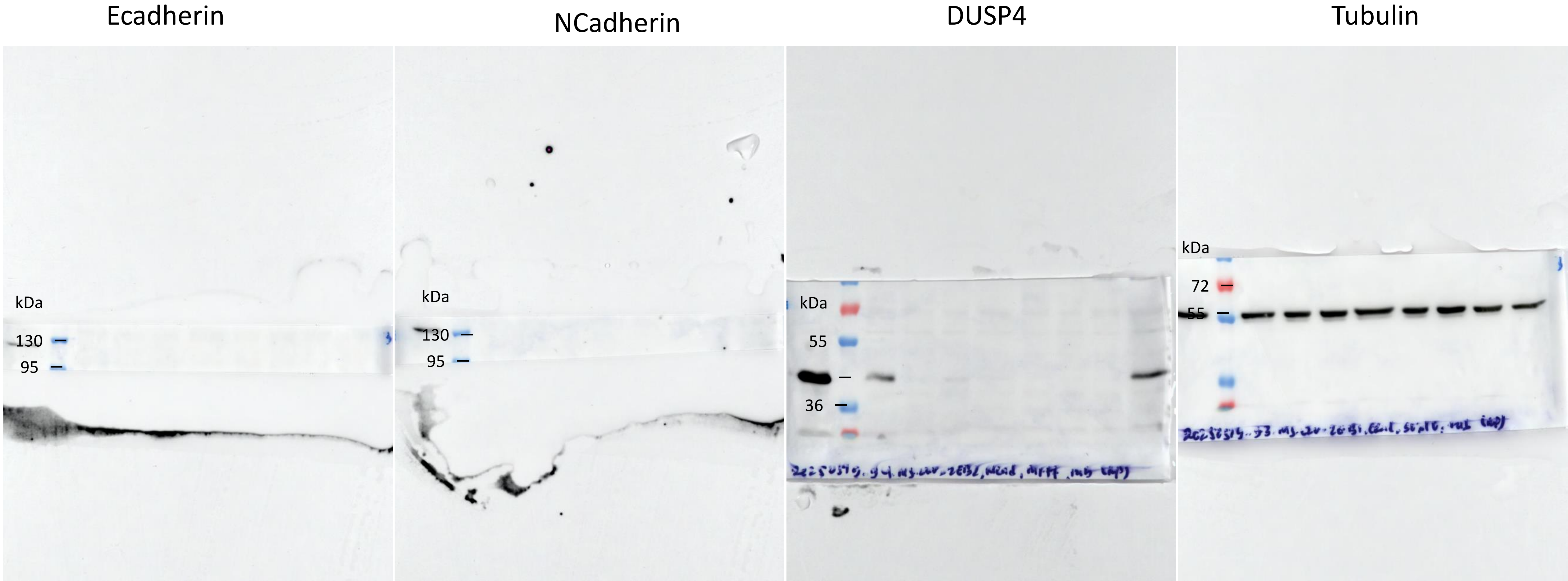

Supp Figure 5B

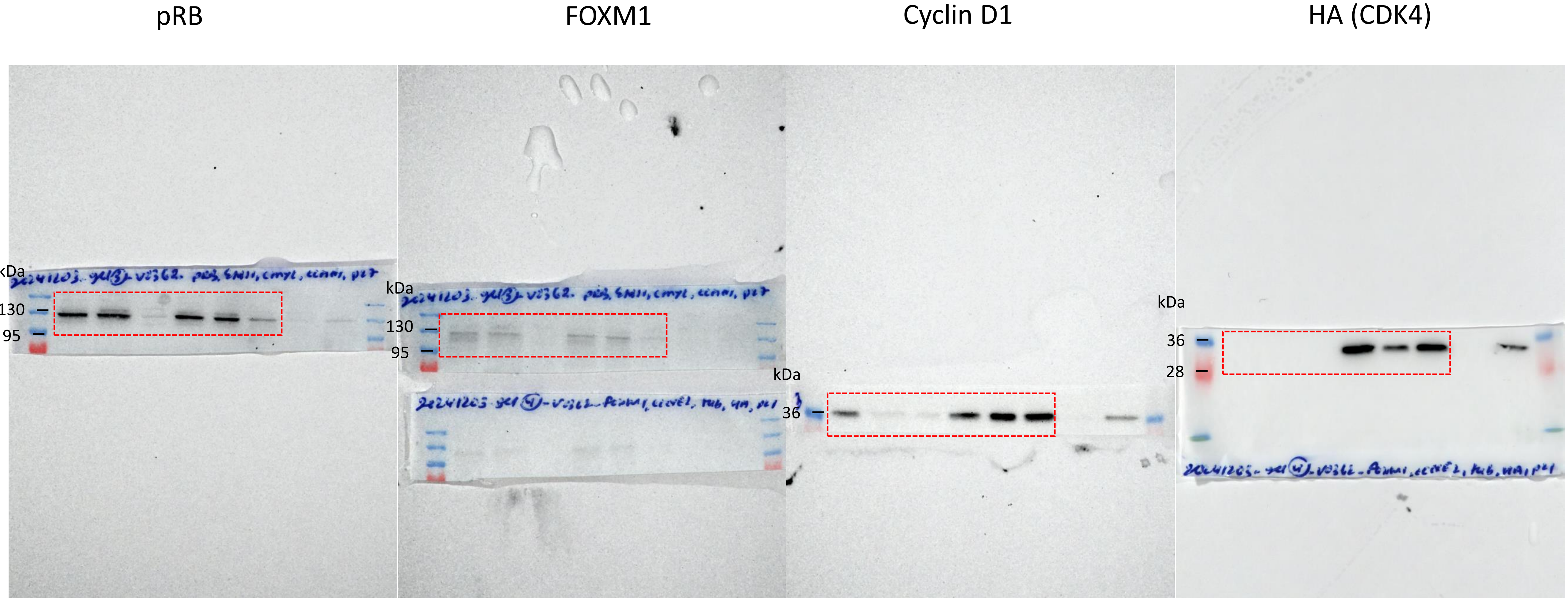

Supp Figure 5B Contd.

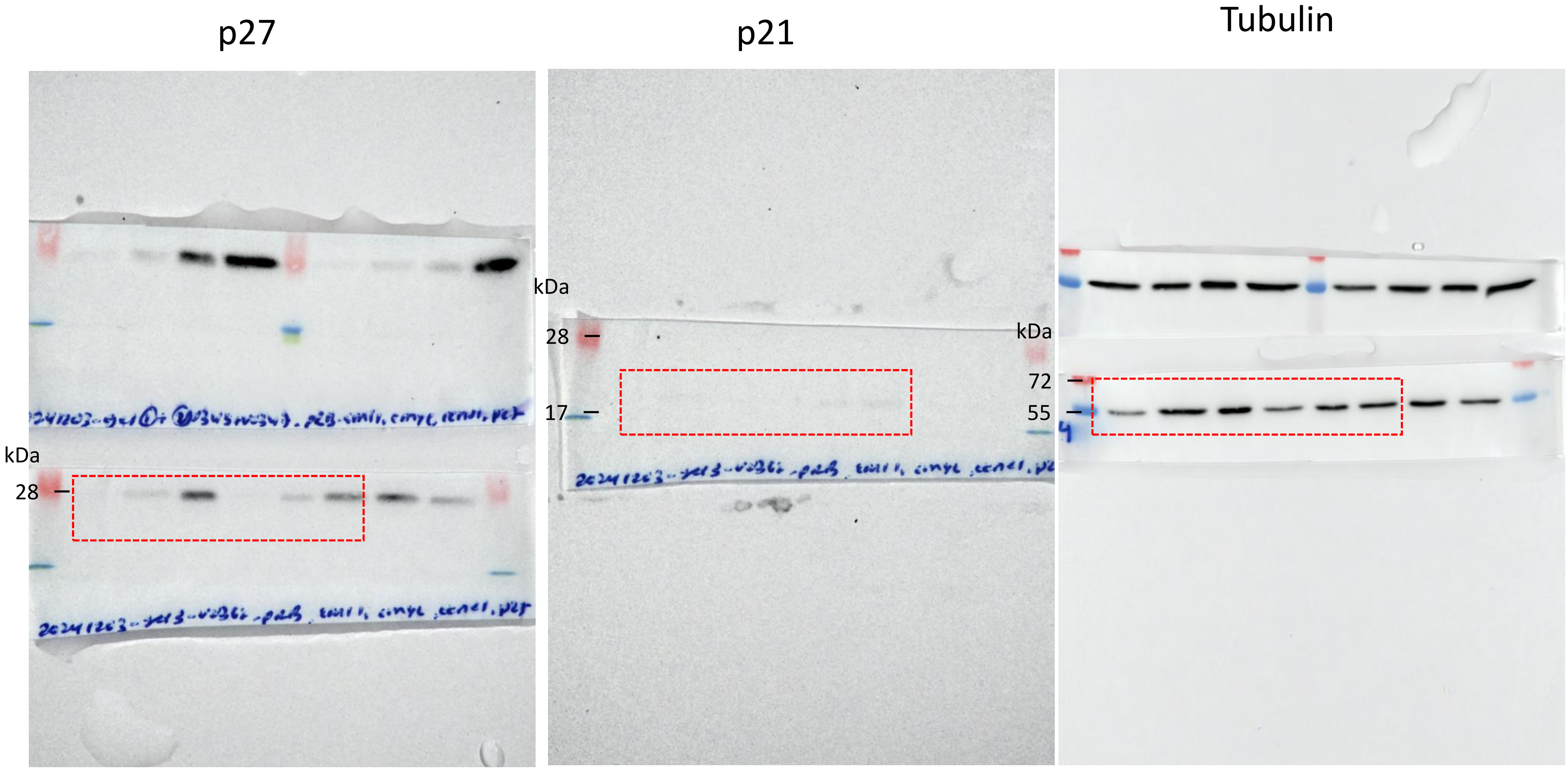

Supplement: Supplementary file 4 — Raw uncut western blots [file 41419_2025_8036_MOESM4_ESM.pdf]
